# Alternative Splicing Events in Tumor Immune Infiltration in Colorectal Cancer

**DOI:** 10.3389/fonc.2021.583547

**Published:** 2021-04-29

**Authors:** Jian-yu Shi, Yan-yan Bi, Bian-fang Yu, Qing-feng Wang, Dan Teng, Dong-ning Wu

**Affiliations:** ^1^ Department of Proctology, Ping Yi People’s Hospital, Linyi, China; ^2^ Department of Proctology, Affiliated Hospital of Shandong University of Traditional Chinese Medicine, Ji Nan, China; ^3^ Department of Basic Pharmacology, College of Integration of Traditional and Western Medicine, Liaoning University of Traditional Chinese Medicine, Shenyang, China; ^4^ Artificial Intelligence and Big Data College, HE University, Shenyang, China; ^5^ Clinical Evaluation Center, Chinese Academy of Chinese Medical Sciences, Beijing, China

**Keywords:** alternative splicing, tumor immune infiltration, prognosis, immunotherapy, colorectal cancer

## Abstract

Despite extensive research, the exact mechanisms involved in colorectal cancer (CRC) etiology and pathogenesis remain unclear. This study aimed to examine the correlation between tumor-associated alternative splicing (AS) events and tumor immune infiltration (TII) in CRC. We analyzed transcriptome profiling and clinical CRC data from The Cancer Genome Atlas (TCGA) database and lists of AS-related and immune-related signatures from the SpliceSeq and Innate databases, respectively to develop and validate a risk model of differential AS events and subsequently a TII risk model. We then conducted a two-factor survival analysis to study the association between TII and AS risk and evaluated the associations between immune signatures and six types of immune cells based on the TIMER database. Subsequently, we studied the distribution of six types of TII cells in high- and low-risk groups for seven AS events and in total. We obtained the profiles of AS events/genes for 484 patients, which included 473 CRC tumor samples and 41 corresponding normal samples, and detected 22581 AS events in 8122 genes. Exon Skip (ES) (8446) and Mutually Exclusive Exons (ME) (74) exhibited the most and fewest AS events, respectively. We then classified the 433 patients with CRC into low-risk (n = 217) and high-risk (n = 216) groups based on the median risk score in different AS events. Compared with patients with low-risk scores (mortality = 11.8%), patients with high-risk scores were associated with poor overall survival (mortality = 27.6%). The risk score, cancer stage, and pathological stage (T, M, and N) were closely correlated with prognosis in patients with CRC (*P* < 0.001). We identified 6479 differentially expressed genes from the transcriptome profiles of CRC and intersected 468 differential immune-related signatures. High-AS-risk and high-TII-risk predicted a poor prognosis in CRC. Different AS types were associated with different TII risk characteristics. Alternate Acceptor site (AA) and Alternate Promoter (AP) events directly affected the concentration of CD4T cells, and the level of CD8T cells was closely correlated with Alternate Terminator (AT) and Exon Skip (ES) events. Thus, the concentration of CD4T and CD8T cells in the CRC immune microenvironment was not specifically modulated by AS. However, B cell, dendritic cell, macrophage, and neutrophilic cell levels were strongly correlated with AS events. These results indicate adverse associations between AS event risk levels and immune cell infiltration density. Taken together, our findings show a clear association between tumor-associated alternative splicing and immune cell infiltration events and patient outcome and could form a basis for the identification of novel markers and therapeutic targets for CRC and other cancers in the future.

## Introduction

Colorectal cancer (CRC) is a highly aggressive form of cancer and is a leading cause of cancer-related mortality worldwide (1). According to the Surveillance, Epidemiology and End Results (SEER) program, 147,950 new cases of CRC were estimated in the USA in 2020. About 50% of patients with CRC suffer from colorectal liver metastasis (2) and a metastasis rate as high as 70-80% has been reported (3). Key aspects of CRC etiology and pathogenesis remain unclear (4). Therefore, in addition to studying the pathogenesis of CRC invasion and metastasis, research on phenotypic regulation is also needed and could facilitate the discovery of precise therapeutic targets or prognostic markers.

The complexity of CRC, which is associated with a series of bio-markers and phenotypes, makes the development of molecular therapies challenging. Genome analysis indicated that CRC tissue exhibited more activated alternative splicing (AS) patterns than adjacent normal samples (5, 6). Several differentially expressed genes (DEGs) for AS in CRC, which could potentially be utilized as predictive or therapeutic targets, have been identified (7, 8). However, the regulatory mechanisms involved in the effects of AS in CRC, especially the effects of AS events on downstream CRC-related pathway regulation, are poorly understood. AS is involved in several processes, including cell growth, invasion, metastasis (8), angiogenesis (9), and chemoresistance in cancers. It is therefore essential to confirm the relationship between specific AS biomarkers and CRC and identify robust AS-related signatures that could serve as targets for CRC treatment. The immune response is an important factor in the occurrence and prognosis of CRC (10). AS of epithelial splicing regulatory protein 1 (ESRP1) was correlated with tumor-associated immune cytolytic activity in malignant melanoma (13). AS plays a crucial role in immune cell diversity and specialization of function. AS-related signatures could therefore be used to identify novel immunotherapeutic targets for CRC.

In the present study, we used bioinformatics to study the relationship between AS and TII characteristics of CRC, and the effect of different AS events on the tumor microenvironment.

## Methods and Materials

### Data Acquisition and Processing

Alternative splicing data from CRC patients were collected from SpliceSeq (http://bioinformatics.mdanderson.org/TCGASpliceSeq), which provides an interactive interface focused on alternative transcripts (11). A list of immune related signatures was obtained from the InnateDB database (https://www.innatedb.ca/), which provides available resources for immunology research (12). We screened the AS data for percent spliced in (PSI) value > 0.75, which represents the association of gene expression and AS events. Overlapping AS events were visualized using an UpSet plot generated using the UpSetR package (13).

We downloaded the CRC genome expression data of 514 patients, including 473 tumor samples and 41 matched normal samples, from the TCGA database (https://portal.gdc.cancer.gov/). We merged the gene expression and clinical profiles using Perl, thereby establishing a genomics and clinical database for further research.

### Identification of AS-Event-Related Prognosis Signatures and Construction of Differential AS-Event Risk Model

The Limma package was utilized to determine the normalization of CRC gene expression, and a differential analysis was applied to screen the abnormally expressed genes in tumor versus normal samples, where the expression differences were characterized by absolute fold change > 2 and *P*-value < 0.05. We then transferred the gene symbol with Entrez ID and performed the Gene Ontology (GO) and KEGG analyses by using the clusterProfiler (14), org.Hs.eg.db (15), enrichplot, and ggplot2 packages with *P*-value<0.05. The Date of GO and KEGG source were updated in 2019. We excluded CRC genomics-clinical data with a futime below 90 d, and performed a univariate Cox regression analysis to obtain the AS related prognostic signatures, choosing the significant signatures with *P* < 0.05, and prepared a Least Absolute Shrinkage And Selection Operator (LASSO) model to reduce the number of variables. We then conducted a multivariate Cox analysis to build up the risk model of differential AS events: the risk model was calculated as Risk score = Ʃ(β_i_ * Exp_i_), where β_i_ represents the weight of the respective signature and Exp_i_ represents the expression value. We could calculate the risk score of each patient in different AS events and classify patients into a high- or low- risk group with the median value as the cut-off value. The reshape2, ggplot2, scales, and cowplot packages illustrated the patients’ vital status distribution and the expression of AS related signatures in the two risk groups of different AS events.

### Assessment of AS Risk Model and Association With Clinical Variables

According to the predictive value of the AS risk model, CRC patients were ranked into high- and low-risk groups. To validate the prognostic ability of the AS risk model in different AS events, we used the timeROC package to construct the ROC curve to show the 3-year OS prediction and determined the predictive effect of AS-related signatures by using the ROC curve. A Kaplan-Meier analysis with a log-rank test was used to assess the survival difference between two risk groups in different AS events. We then performed univariate and multivariate Cox regression analyses to determine the relationships between the risk score and clinical features consisting of TNM stages or pathological stages. We used the Wilcoxon rank-sum test to compare the two groups, and the Kruskal-Wallis test for three or more groups, *via* the survival and forest plot packages.

### Construction and Assessment of the Immune Risk Model in CRC

After normalization of CRC gene expression data, we identified the DEGs in tumor versus normal samples, characterized by absolute fold change > 2 and *P*-value < 0.05. We then obtained intersecting immune related signatures from an immune gene list obtained in the InnateDB database. To investigate the hub immune genes for predicting the prognosis of CRC, we merged DEG expression and clinic profile by using Perl.

We conducted univariate Cox regression analysis to the immune DEGs and obtained immune prognostic signatures of patients with CRC. In order to reduce the number of variables, we selected the significant signatures with *P* < 0.05 and prepared a LASSO model. Finally, according to the result of a multivariate Cox analysis, we constructed the immune risk model calculated as TII Risk score = Ʃ(β_i_ * Exp_i_), where β_i_ represents the weight of the respective signature and Exp_i_ represents the expression value. We were then able to classify patients with CRC into high- or low-immune risk groups with the median value as the cut-off data.

We verified the predictive value of the risk model by using clinical survival outcomes and applied the ROC curve to show the 3-year OS prediction *via* the timeROC package. We conducted a Kaplan-Meier analysis with a log-rank test for survival comparison between the low- and high-immune risk groups. Finally, univariate and multivariate Cox regression analyses provided the relationships between the immune risk score and the clinical features consisting of TNM stages or pathological stages *via* the survival and forestplot packages.

### Tumor Immune Infiltration in Patients With CRC

We evaluated the associations between immune risk score and TII base in the TIMER database, which is a public resource for the systematic analysis of immune infiltrates in multiple malignancies. Form TIMER, we could obtain the abundances of six immune infiltrates, including B cells, CD4^+^ T cells, CD8^+^ T cells, neutrophils, macrophages, and dendritic cells according to statistical and pathological estimation results. With the CRC-TII information from TIMER, we evaluated the associations between immune risk score and immune infiltration cells by using the *P*-value calculated from Pearson’s correlation coefficient.

### Validation of Survival Correlation Between Prognosis Signature and Tumor-Infiltrating Immune Cells in Patients With CRC

To identify the relationship between TII and different AS events, we evaluated the correlation between TII and AS in two aspects. First, we explored the association between TII and AS risk. Based on the risk score of OS prognosis signature, we conducted a two-factor survival analysis of 379 patients, thus determining the relationship between TII risk and risk of different AS events. We then investigated the effect of different AS events on TII cells in patients with CRC. According to TIMER, we assigned 6 different types of TII cell density values in 379 patients with CRC and divided these samples into high- and low-risk groups based on the AS risk model. We performed the log-rank test to evaluate the distribution of the 6 different types of TII cells in the high-risk and low-risk groups in seven different AS events and in total. Finally, we illustrated the *P-*value (*P*-value < 0.05 was considered statistically significant) of the difference in infiltrating immune cells in the AS-high-risk and AS-low-risk groups in a CRC tumor microenvironment by using a pheatmap package. We then visualized the TII cell landscape of *P*-value < 0.05 by using the survival package.

### Statistical Analysis

All data were used to determine independent prognostic factors, which could predict patient survival status by using the R package (R software version 3.6.3). All statistical analyses were performed using the IBM SPSS 18.0 program. The Student *t*-test (for equal variances) was used to analyze data, and *P*-values (two-sided test) < 0.05 were considered significant.

## Results

### Identification of Seven AS Events Prognostic Related Signatures in CRC

**Figure 1:**
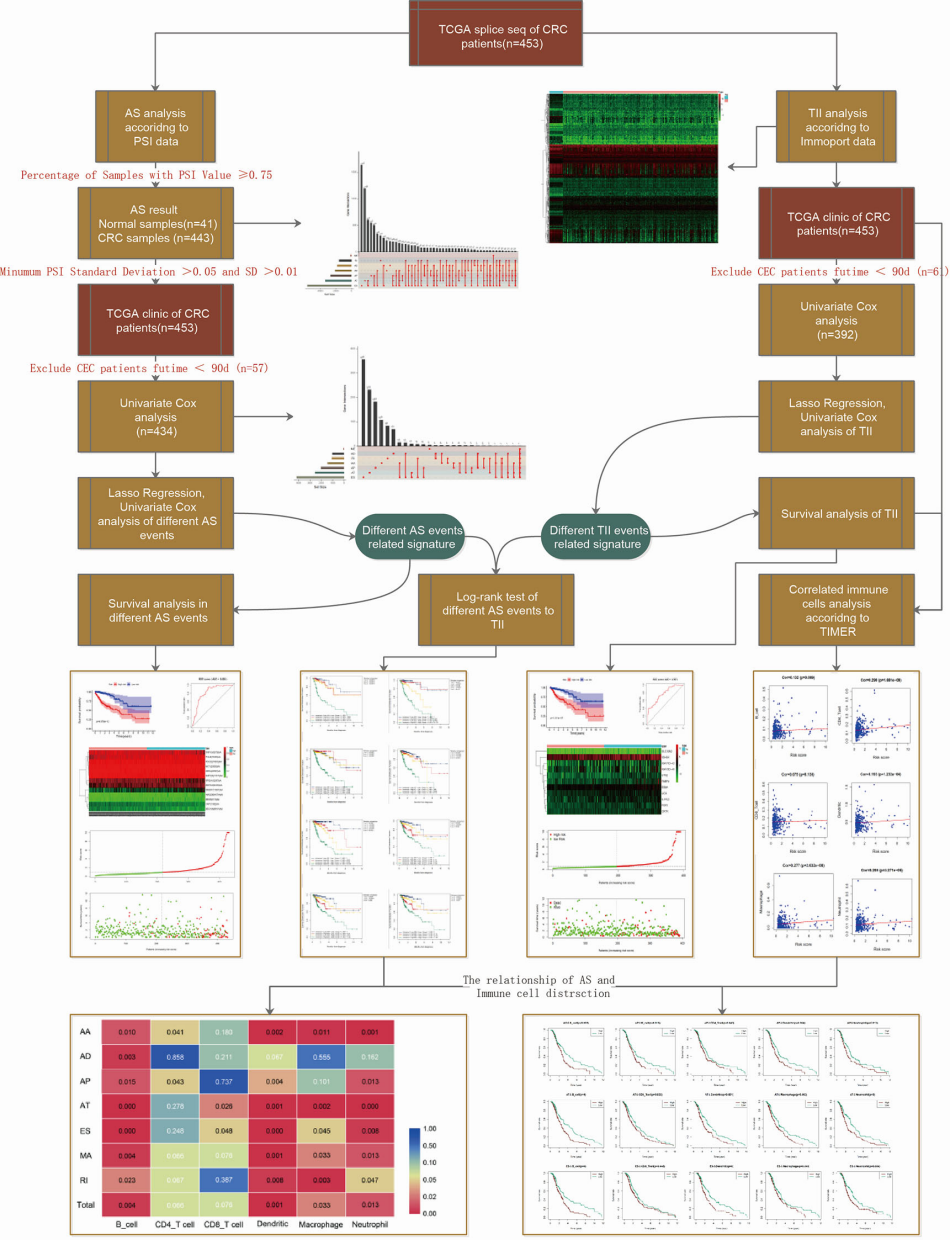
Schematic representation of the work-flow.

**Figure 2:**
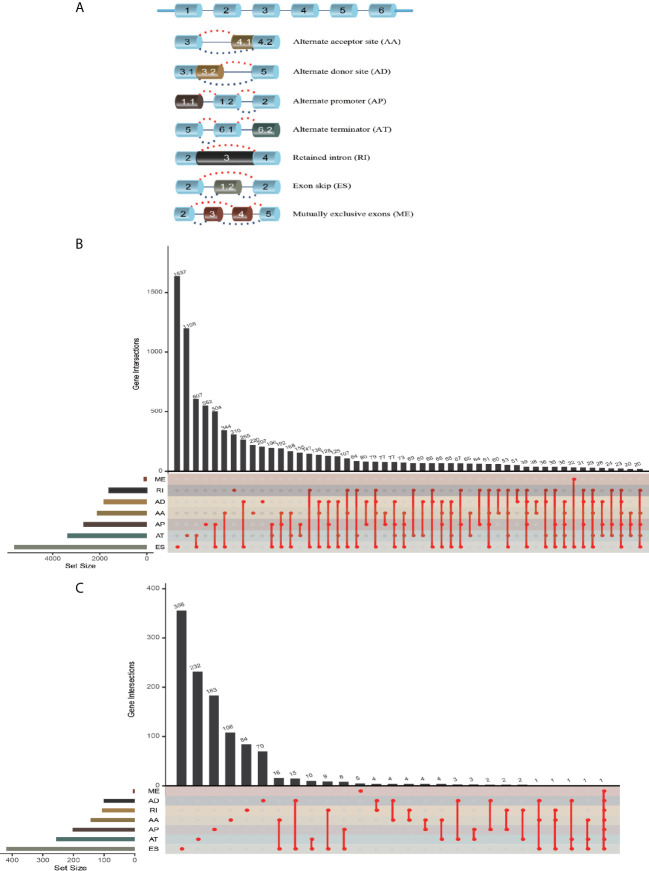
AS event profiling in CRC. **(A)** Representative model of seven different AS types. **(B)** UpSet plot of interactions between different AS types in CRC (n = 452). **(C)** UpSet plot of different survival-associated AS types obtained a univariate Cox regression in CRC.

The workflow of this study is shown in **Figure 1**. We obtained the profiles of AS events/genes for 484 CRC patients, consisting of 473 CRC tumor samples and 41 corresponding normal samples, from the TCGA database. We excluded patients without clinical data, and the complete information is shown in **Table 1** (n = 452). We screened the data by minimum PSI standard deviation > 0.05 and SD > 0.01, and excluded patients with insufficient clinical data (including futime below 90 d). The seven AS events classified in the study (**Figure 2A**) were: Alternate Acceptor site (AA), Alternate Donor site (AD), Alternate Promoter (AP), Alternate Terminator (AT), Exon Skip (ES), Mutually Exclusive Exons (ME), and Retained Intron (RI). The interaction between genes and the different AS types was displayed in an Upset plot (see **Figure 2B**). We detected 22581 AS events in 8122 genes; ES (8446) was the most common AS event, and ME (74) was the rarest.

**Table 1:** Baseline characteristics of 452 CRC patients included in this study.

Variables	Count	Percentage (%)
**Age** (Mean ± SD)	67.26 ± 13.02
**Follow-up** (y)	2.05 ± 1.98
**Status**		
Alive	88	19.47
Dead	364	80.53
**Gender**		
Male	238	52.65
Female	214	47.34
**AJCC-T**		
T1	10	2.21
T2	77	17.04
T3	308	68.14
T4	56	12.39
Tis	1	0.22
**AJCC-N**		
N0	269	59.51
N1	103	22.79
N2	80	17.70
**AJCC-M**		
M0	334	73.89
M1	62	13.72
MX	49	10.84
Unknow	7	1.55
**Pathological stage**		
I	76	16.81
II	178	39.38
III	125	27.65
IV	62	13.72
Unknow	11	2.43
**Grade**		
G1	–	–
G2	–	–
G3	–	–
G4	–	–
Unknow	452	100.00

AJCC, American Joint Committee on Cancer.

After merging the clinical data by Perl, we conducted a univariate Cox proportional hazards regression analysis and detected 1468 survival-associated AS events in 1132 genes. The interaction between these genes and different AS types is shown in **Figure 2C**; seven AS events were associated with OS in patients with CRC, ES (463) was the most commonly occurring AS event, and ME (5) was the rarest. A volcano plot (see **Figure S1A**) was used to identify AS-related DEGs (*P*-value < 0.05 and |log FC| ≥ 1.95) in CRC, and to evaluate the prognostic value of these DEGs. The top 20 signatures in the seven AS types are shown in **Figures S1B–H**.

To determine the biological roles of the selected signatures in AS processing, the KEGG pathway (**Figures S2A–D** and **Table S1**) and GO categories (**Figures S2E–H** and **Table S1**) were established. Significant KEGG pathways of the AS events included steroid biosynthesis (16), ubiquitin-mediated proteolysis (17), transcriptional misregulation in cancer, and RNA transport. The GO functional analysis indicated that a series of GO functions, such as coenzyme metabolic process regulating GTPase activity, positive regulation of GTPase activity, proteasome-mediated ubiquitin-dependent protein catabolic process, proteasomal protein catabolic process, and histone modification, take part in the mRNA splicing process. In summary, the selected signatures participated in AS events in the CRC-related biological process.

### Establishment of Risk Score and AS Prognosis Model Assessment

**Figure 3:**
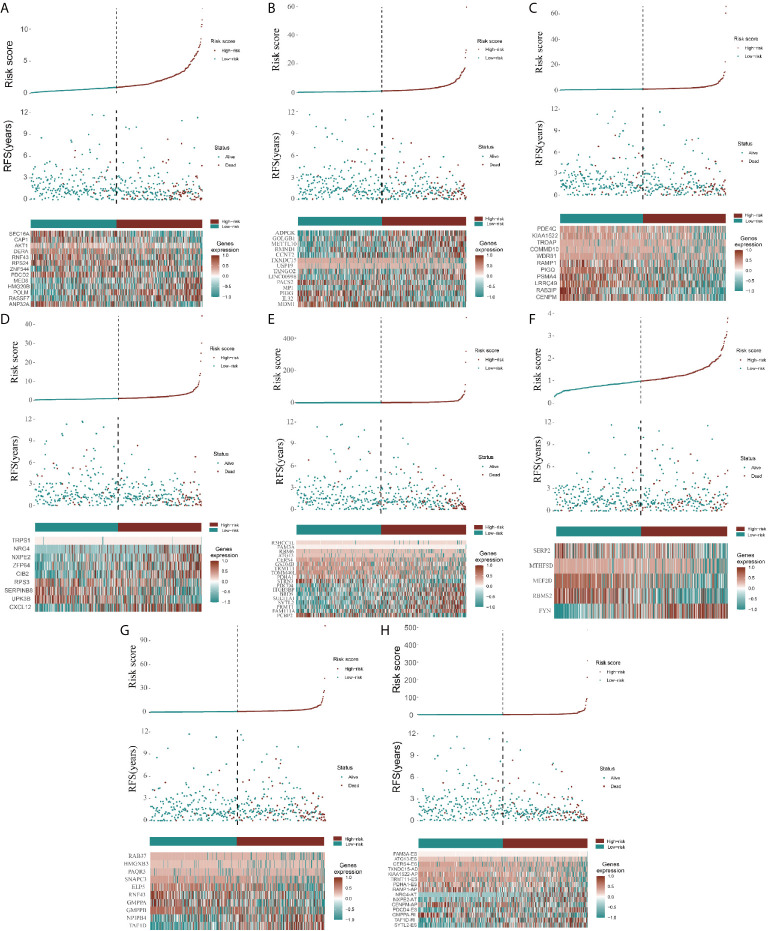
Construction of AS model and distributions. **(A–H)** represents the AS events AA, AD, AP, AT, ES, ME, RI, and total events, respectively. In each figure, the top section shows patient survival data sorted according to the risk levels, the middle section shows the risk score distribution curve, and the bottom section shows the differences in levels of identified hub immune signatures between high-risk and low-risk groups as a heatmap plot.

**Figure 4:**
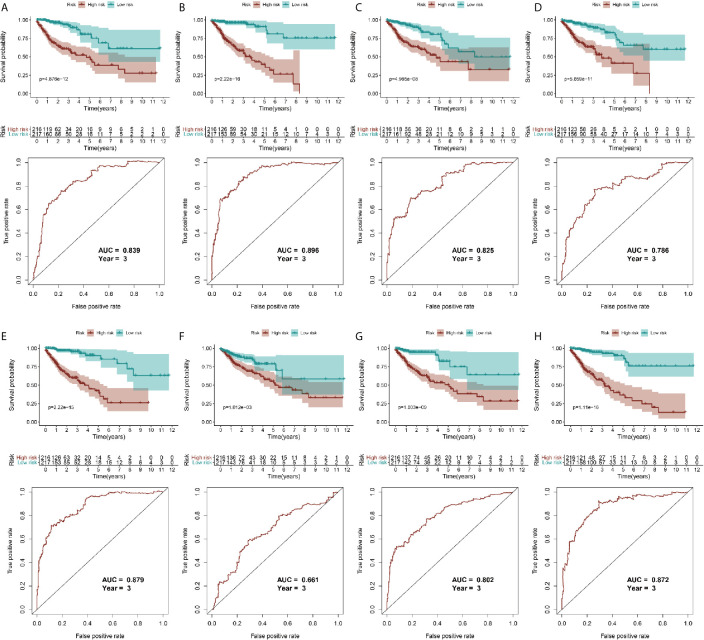
Validation of AS model with survival results. **(A–H)** represents the AS events AA, AD, AP, AT, ES, ME, RI, and total events, respectively. In each figure, the top section shows Kaplan-Meier curves of prognostic signatures in patients with CRC, and the bottom section shows the ROC curves of prognostic predictors in patients with CRC at 3 years.

Because the AS-related signatures showed differential levels in tumors and normal samples, we merged these DEGs with survival data and used univariate Cox analysis to obtain the prognostic signature in different AS events with *P* < 0.05, to screen prognostic genes in different AS events. We then performed a Lasso regression to find the most significant prognostic signatures according to the univariate Cox results (**Figure S3**) and conducted a multivariate Cox regression analysis. Finally, we obtained a list of prognostic signatures in different AS events (**Table S2**) and calculated the risk score according to the weight of each gene. We could therefore classify the 433 patients with CRC into low-risk (n = 217) and high-risk (n =216) groups by using the median risk score in different AS events. As shown in **Figure 3**, patients with a high-risk score had more survival risks. Differentially expressed levels of hub genes in seven AS events in the low-risk and high-risk groups are also shown in the heat map in **Figure 3**. The ROC plots of different AS events are presented in **Figure 4**. The AUC value ranged from 0.661 (MA) to 0.896 (AD), indicating effective prediction accuracy of the risk model for the prognosis of CRC. As shown in **Figure 4**, the Kaplan-Meier analysis for the log-rank test demonstrated that in different AS event cohorts, patients with high risk scores were associated with poor OS (*P* < 0.001).

**Figure 5:**
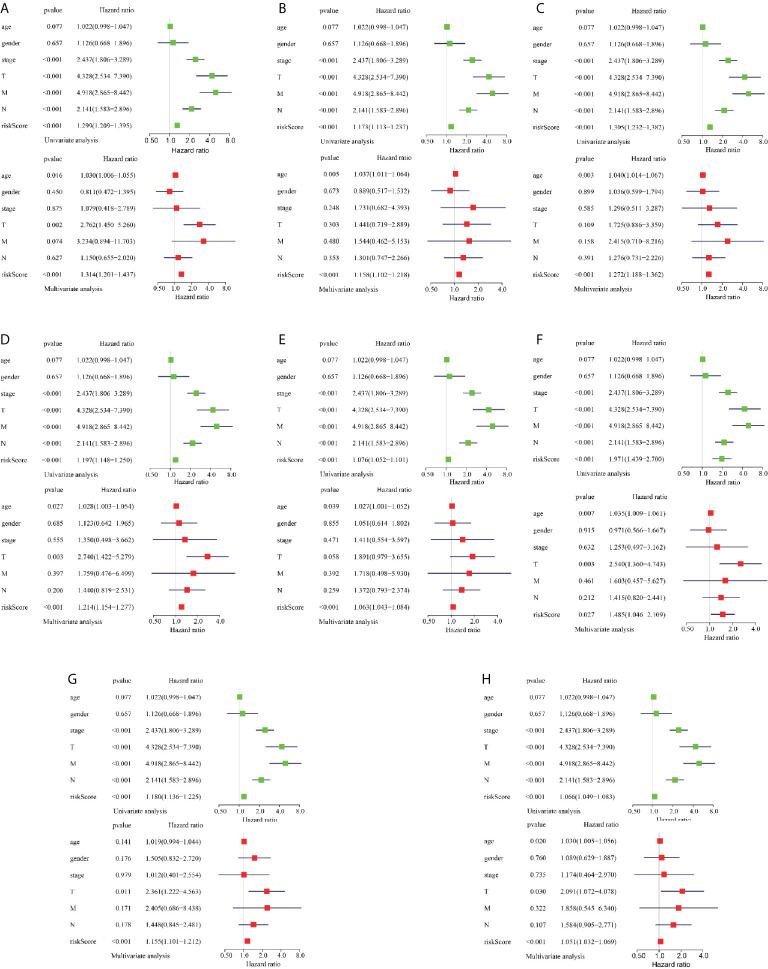
Assessment of AS risk model in predicting CRC prognosis. **(A–H)** represents the AS events AA, AD, AP, AT, ES, ME, RI, and total events, respectively. Forest plot visualizing hazard ratios of significant survival-related clinical pathological parameters including age, gender, stage, and pathological stage T, M, and N obtained by univariate (top-green point) and multivariate (bottom-red point) Cox regression analysis.

To reveal the correlation between CRC prognosis and clinical characteristics, we performed univariate and multivariate survival tests to evaluate the prediction ability of the risk model for different clinical pathological parameters including age, gender, cancer stage, and pathological stage-T, M, and N (**Figure 5**). The univariate survival analysis indicated that the risk score and the cancer stage and pathological stage-T, M, and N were closely correlated to the prognosis of patients with CRC (*P* < 0.001) in different AS event cohorts. The multivariate survival analysis indicated that the risk score was the only independent prognostic indicator with significant differences (except MA; *P* = 0.027, all the other cohorts *P* < 0.001), indicating that, in comparison with the other characteristics, the risk score exhibited the greatest potential for clinical application.

### Establishment of Risk Score and TII Prognosis Model Assessment In the CRC Immune Microenvironment

TII profiles from patients with CRC were obtained from the TCGA database, and samples with insufficient clinical data were excluded (**Table 1**). We then merged the clinical and transcriptome data from the databases, after excluding patients with futime below 90d, and included 392 tumor samples for subsequent study. As shown in **Figure S4A**, we identified 6479 DEGs from transcriptome profiles with |log fold change| > 1 and false discovery rate (FDR) < 0.05. From the InnateDB database, we obtained a list of 2498 immune-related genes, and intersected 468 differential immune-related signatures. The expression of these signatures in CRC samples is shown as a heat map (see **Figure S4B**).

**Figure 6:**
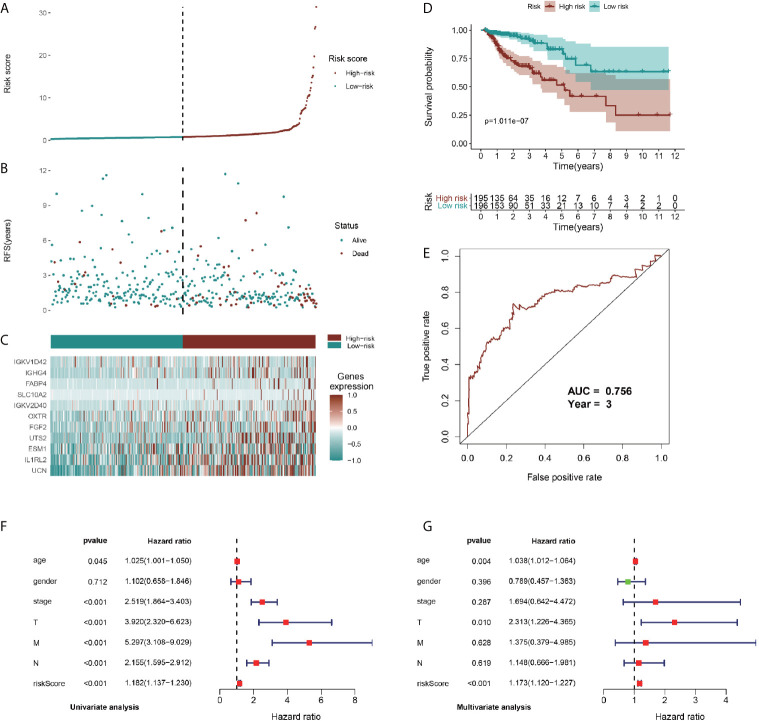
Identification, construction, and validation of the TII prognostic model system in CRC. **(A, B)** Patient survival data in the two risk groups. **(C)** Heatmap plot showing differences in levels of identified hub immune signature between high-risk and low-risk groups. **(D)** Kaplan-Meier curves of prognostic signature for CRC patients. **(E)** 3-year ROC curves for prognostic predictors. Univariate **(F)** and multivariate **(G)** Cox regression analyses were conducted to assess the immune-related signatures and clinic characteristics using Forest plots.

To identify prognostic genes among the selected immune-related signatures, we performed univariate Cox analyses and screened 20 TII prognosis signatures (**Figure S4C**). As shown in **Figures S4D, E**, we optimized the models by Lasso regression analyses. To build up the *optimal* immune-related risk scoring model, based on clinical OS data, we conducted multivariate Cox analyses and identified 11 hub immune genes, with the risk scoring model according to the weight of each gene (**Table S3**). The patients enrolled in this study were classified into low-risk (n = 196) and high-risk (n = 195) groups. As shown in **Figures 6A, B**, the high-risk group exhibited a higher survival risk. The hub gene expression data of the two groups are shown in **Figure 6C**, and OS results are shown in **Figure 6D**. The Kaplan-Meier analysis results indicated that, compared with the low-risk group, the high-risk group was associated with a poorer OS (*P* < 0.001), and the 3-year area under curve (AUC) was 0.756, which confirmed the prediction accuracy of the risk score for CRC prognosis (**Figure 6E**). To verify the predictive ability of the risk model, we performed univariate and multivariate survival tests to evaluate the predictive ability towards different clinical pathologies, including age, gender, cancer stage, and pathological stage-T, M, and N. As shown in **Figure 6F**, the risk score and the cancer stage, and pathological stage-T, M, and N were closely correlated to the prognosis in patients with CRC (*P* < 0.001) based on the univariate survival analysis result. Multivariate survival analysis (**Figure 6G**) showed that the risk score (*P* < 0.001) and stage-T (*P* = 0.010) were the only independent prognostic indicators with significant differences.

**Figure 7:**
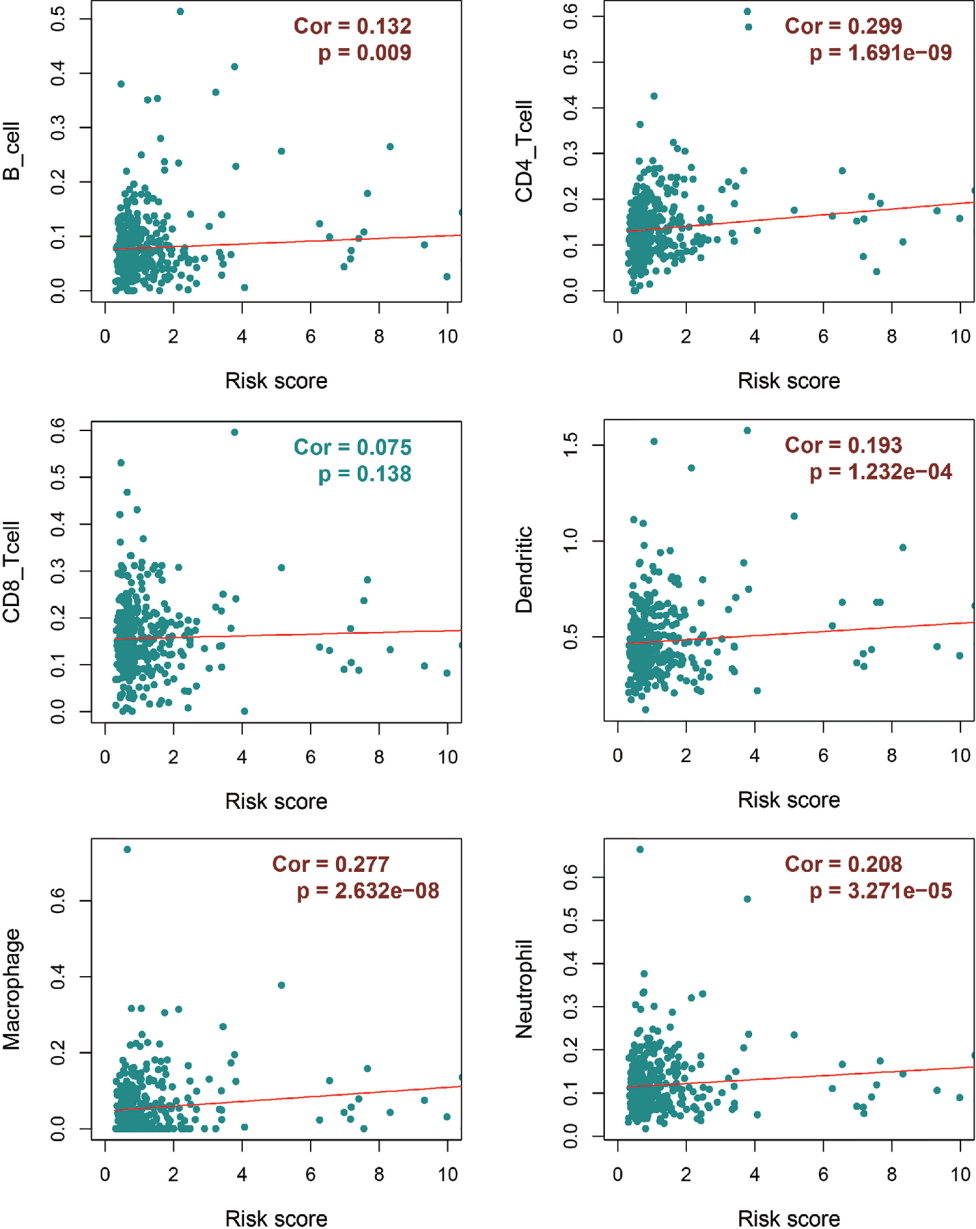
Integrative analysis of TII risk level and six types of tumor-infiltrating immune cells.

Regarding the dominant roles of TII cells in the CRC microenvironment, we integrated the immune-related signatures combined with immune infiltrates. Based on the TIMER database, we evaluated the distribution of 6 types of TII cells in the TII low-risk and TII high-risk groups. As shown in **Figure 7**, the high-risk groups exhibited a lower B-cell, CD4_T cell, dendritic, macrophage, and neutrophil concentration (*P* < 0.01) than that in the low-risk groups.

### Relationship Between Different AS Events and the TII Risk Model

Abnormalities in TII cells are accompanied by cancer initiation and progression (18) in the tumor microenvironment. In the present study, the higher TII risk score represents a poorer OS, higher clinic risk, and lower infiltration of immune cells. To elucidate the relationship between different AS events and TII, we evaluated the correlation of the AS and TII risk models, and comprehensively analyzed the relationship of different AS events with immune infiltrates.

**Figure 8:**
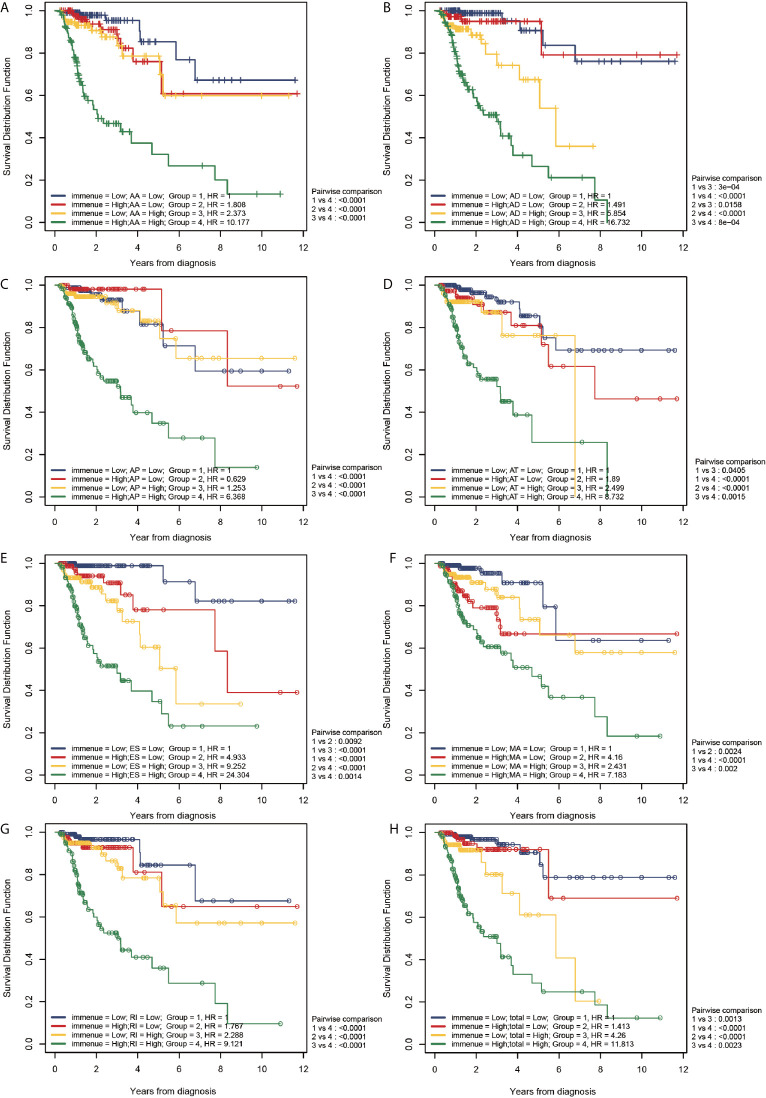
Correlation between the TII risk and AS events. **(A–H)** represents the AS events AA, AD, AP, AT, ES, ME, RI, and total events, respectively. We conducted a two-factor analysis to identify the risk of different AS events with TII risk in 4 groups: Group 1 (immune risk = Low; AS risk = Low), Group 2 (immune risk = High; AS risk = Low), Group 3 (immune risk = Low; AS risk = High), and Group 4 (immune risk = High; AS risk = High).

**Figure 8** shows the correlation of TII risk score and the risk score for different AS events by comparing four different risk groups, namely group 1: patients with low TII and low AS score, group 2: patients with high TII and low AS score, group 3: patients with low TII and high AS score, and group 4: patients with high TII and high AS score.

According to the results of the log-rank analysis, the following conclusions could be drawn:

1. In all AS events, group 1 exhibited a significantly higher OS compared than group 4 (*P* < 0.001). In higher AS risk groups (groups 3 and 4), the patients with a higher TII risk had a significantly poor prognosis compared to patients with low TII risk (*P* < 0.01).
2. In all AS events (except MA), patients with higher TII risk and AS risk (group 4) had a significantly poorer OS compared to patients in groups 1-3 (P < 0.01). However, in MA events, patients in group 4 exhibited a significantly lower OS than patients with a lower TII risk (groups 1 and 3; *P* < 0.001), and patients with higher TII risk (groups 2 and 4) exhibited a similar OS result. These results confirmed that for MA events, in the low TII risk group, the prognosis in patients with CRC was closely correlated to the AS event risk score, and there was a limited relationship between the OS and AS event risk score in high TII risk groups.

3. As shown in **Figure 8**, groups 2 and 3 exhibited a similar OS result (*P* > 0.05), except for AD events (**Figure 8B**), indicating that all AS events (except AD) exhibited a similar OS risk compared with TII. A further comparison revealed that in AD events, high and low TII risk groups exhibited a similar OS in the low AD risk groups (groups 1 and 2); and with the increase of AD risk, patients suffered a decrease of OS with the increase of TII risk. For instance, in group 3 (low TII and high AS), patients exhibited a significantly lower OS than patients in groups 1 and 2 (*P* < 0.001), and with the increase of TII risk score, the patients with high AD risk showed a further decrease in OS result (group 3 vs group 4, *P* < 0.001).

4. The patients in group 1 (lower AS and TII risk) were not associated with a higher overall survival compared with patients in groups 2 and 3 (*P* > 0.05), except towards ES events. As shown in **Figure 8E**, compared with group 1, group 2 (*P* = 0.0092) and group 3 (< 0.001) exhibited a significantly lower OS, indicating that in ES events, lower AS and TII risk are associated with the OS of patients and a good prognosis.

### The Relationship Between Different AS Events and TII Cell Infiltration in the Tumor Microenvironment

**Figure 9:**
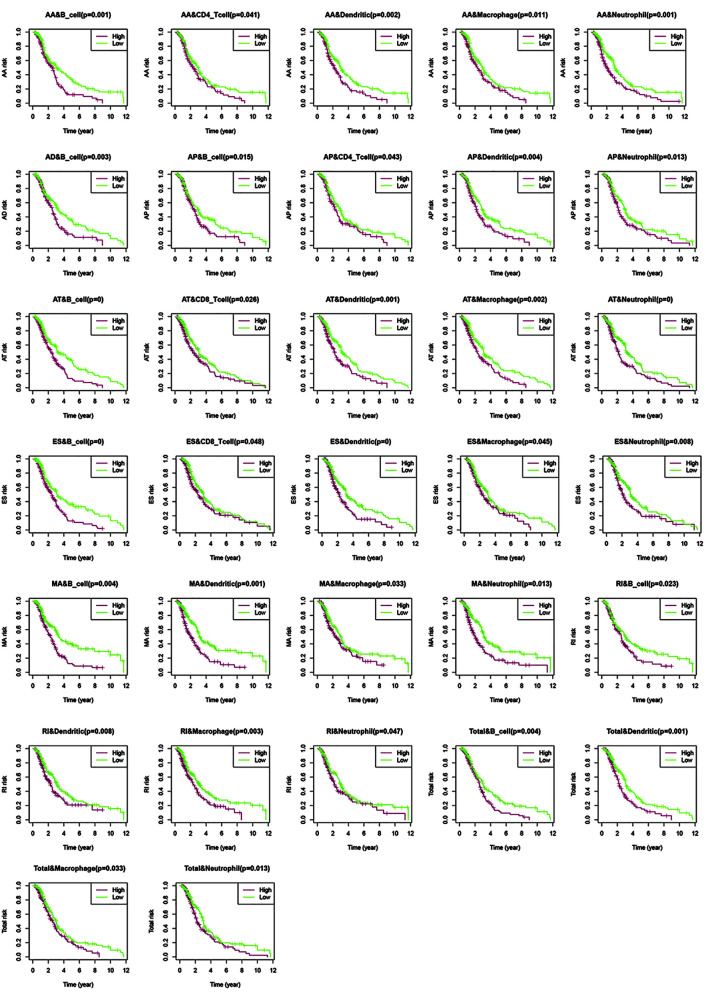
Assessment of AS risk in relation to six immune infiltration cell types in CRC. After determining the correlation between low- and high-risk of different AS events in each cell type, samples with a calculated *P*-value < 0.05 were visualized.

**Figure 10:**
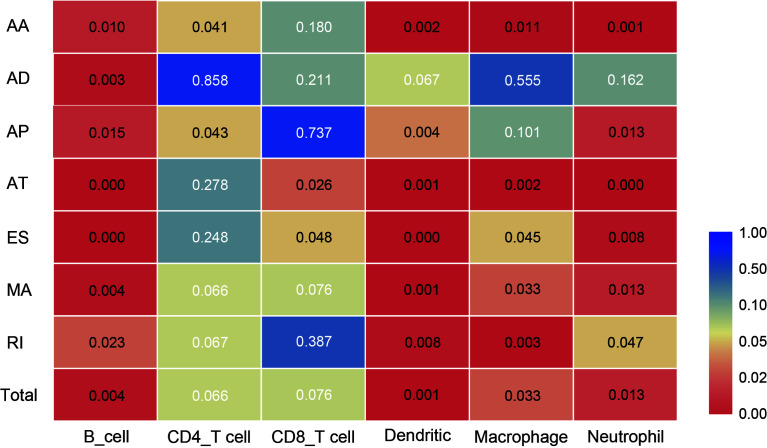
Heat map showing differences in the infiltration of six immune cell types in the low-risk and high-risk groups of different AS events. Colors from blue to red represent high to low *P*-values, and yellow represents *P* = 0.05.

Previous reports have indicated that different AS events are associated with different TII characteristics in patients with CRC. To identify the immune context in different AS events, we used a TIME database to estimate the proportions of six distinct immune cell types in patients with CRC. We then studied the correlation between low-risk and high-risk of different AS events for each cell type. We excluded samples with a calculated *P*-value > 0.05 (**Figure 9**) and found that:

1. The proportions of B cells were significantly higher in the low-risk group than in the high-risk group (*P* < 0.05). Dendritic cells and Neutrophils exhibited a similar trend (*P* < 0.05), except in AD events (*P >* 0.05).

2. In all the AS events, the proportion of CD4T cells exhibited significant differences in AA and AP events, while CD8T cells exhibited significant differences in AT and ES events. These results suggested that CD4T cells and CD8T in the immune infiltrate of CRC appeared to have a limited response to AS events.

3. Compared with other events, only AD events were associated with B cell immune infiltration (*P* < 0.05). AT and ES events had a strong effect on all immune cells except CD4T cells.

4. All the above results show that in the tumor microenvironment, B cells, dendritic cells, macrophages, and neutrophils were powerfully associated with AS, and AS events had a limited effect on the infiltration of CD4T cells and CD8T cells. The statistical results between TII cell distribution and AS risk score are shown in **Figure 10**.

## Discussion

CRC is a leading cause of cancer-related deaths worldwide (4,20). Several DEGs have been identified as predictive and therapeutic signatures in CRC. The pathogenesis and development of CRC is a complex process involving multiple pathways and phenotypes. AS and TII play an important role throughout the development of CRC. TII illustrates the macroscopic characteristics of the tumor microenvironment, while AS represents specific processes of cancer progression (19).

AS is a process wherein a single pre-mRNA is variably spliced into unique transcripts, which causes diversity in the downstream transcriptome and proteome, and could contribute to the establishment of cancer sites. Abnormal AS events are often closely related to cancer progression, and specific AS events could predict tumor occurrence (20). AS-related genes have been studied as potential prognostic signatures in pancreatic (21), esophageal (22), and lung (23) cancer. Landscape analysis of AS events and correlation with specific tumor phenotype and progression could yield prognostic, predictive, and therapeutic clinical signatures (24).

During radioactive tracer treatment of lung cancer cells, SRSF1 knockdown could sensitize cancer cells to irradiation via regulation of PTPMT1 splicing (25). Luo (26) identified SRSF2 as a regulator of alternative splicing events, which increased cell proliferation and tumorigenic potential by controlling variant expression. Georgilis (28) has suggested that the senescence-associated secretory phenotype regulated by PTBP1 is a bridge between AS events and immuno-oncology. Li (27) evaluated the correlation between cancer-associated AS events (CASEs) and the immune microenvironment in head and neck squamous cell carcinoma (HNSC) and demonstrated that CASE parent genes were significantly enriched in pathways related to HNSC and the TII microenvironment. Transcriptomics studies of CRC have shown that different AS events in mRNA are key factors in CRC tumor formation and deterioration (28).

Tumor cells often locally secrete a series of cytokines that facilitate the interaction between the tumor cells and the extracellular matrix, thereby forming a suitable environment for cellular growth and proliferation (29). In a TII microenvironment, where the immune system could protect tumor cells from the immune barrier, cancer immune-editing and resistance could cause “immune escape” (30), thus promoting the proliferation, invasion, and metastasis of tumor cells. CRC research focusing on immunology demonstrated that macrophages, natural killer cells, dendritic cells, immature and memory lymphocytes, mast cells, B cells, and effector T cells were closely correlated to the TII environment (31, 32). We, therefore, hypothesized that the correlation between AS events and TII, and systematic research of AS-TII could yield potential prognostic and therapeutic targets in patients with CRC.

In the present study, we identified 96 signatures related to AS events, where 18 signatures, including RNF43, KIAA1522, NRG4, CERS4, and TRMT11, intersected in 7 AS event types. Based on the prognosis results, we selected CERS4, KIAA1522, NRG4, TRMT11, SYTL2, PDCD4, ATG13, CENPM, FAM3A, NXPE2, GMPPA, TXNDC15, TAF1D, PDHA1, and RAMP1 as AS-event-related signatures for predictive prognosis in CRC. CERS4 (ceramide synthase 4) generates ceramide, which binds to Smad7 and limits the aggregation of TGF-β receptor in the primary cilia, thereby blocking tumor cell migration. CERS4 expression was down-regulated in metastatic (late stage) head and neck squamous cell carcinoma (HNSCC), renal cell carcinoma, and melanoma (33), indicating that activation of CerS4/ceramide-Smad7 could target and inhibit tumor cell migration and invasion. KIAA1522 is a coding gene, which is significantly up-regulated in lung and breast cancer (34). In esophageal squamous cell carcinoma samples (35), the CpG island in the promoter region of the KIAA1522 gene exhibited high-frequency methylation, which suggests that KIAA1522 is an oncogene that promotes cancer cell proliferation (36). The epidermal growth factor (EGF) family is closely associated with cancer (37); HER4 (ErbB4) is expressed in a series of isoforms owing to AS (38). HER4 was down-regulated in all isoforms of gastric cancer cells and could be an anti-carcinogenic target (39).

In androgen deprivation treatment of advanced prostate cancer, TRMT11 (tRNA methyltransferase 11) exhibited the strongest association with time to therapy failure (P < 0.001; FDR0.008; (40). In ovarian cancer, with over-expression of SYTL2 (synaptotagmin like 2) mRNA, the methylation rate in specific CpG sites of the SYTL2 promoter decreased, which significantly promoted migration and invasion of ovarian cancer cells (41). As a novel tumor suppressor gene, programmed cell death 4 (PDCD4) could regulate multiple signal transduction pathways of colon cancer (42), thus inhibiting tumor progression at the transcriptional and translational levels (43, 44). DTL (Denticleless E3 ubiquitin protein ligase homolog) and SKP2 (S-phase kinase-associated protein 2) could degrade cancer progression *via* PDCD4 ubiquitination (45). Autophagy-related protein 13 (ATG13) phosphorylation is a crucial bioprocess in autophagy in epithelial ovarian cancer (EOC) (46). Over-expression of ATG13 caused by binding with CircMUC16 magnified autophagy and exacerbated EOC invasion and metastasis. Centromere protein M (CENPM), an important regulator in P53 signaling and cell cycle pathways, was over-expressed in hepatocellular carcinoma tissues (47), and could be an immunotherapy target. NXPE2 (neurexophilin and PC-esterase domain family member 2) could be a predictive signature for diffuse-type gastric cancer heterogeneity (48). GMPPA (GDP-mannose pyrophosphorylase A) mutations were utilized for predicting lung adenocarcinoma, lung squamous cell carcinoma, and small cell lung cancer (49). Down-regulation of PDHA1 (pyruvate dehydrogenase E1 subunit alpha 1), an important enzyme complex in cancer metabolism, could promote glycolysis and accelerate the progression of gastric cancer cells (50). Moreover, PDHA1 knockout significantly accelerated glycolysis, increased the consumption of glucose and glutamine, and suppressed oxidative phosphorylation, thereby causing the Warburg effect, in the KYSE450 esophageal cancer cell line (51). In Ewing’s sarcoma (EwS) clonogenic/spheroidal growth and tumorigenicity (52), RAMP1 (receptor activity modifying protein 1) is a crucial receptor in the assembly of the secretory neuropeptide calcitonin-related polypeptide beta (CALCB). Knockdown of RAMP1 in prostate cancer cells blocks the expression of MAP2KI (Dual specificity mitogen-activated protein kinase 1) and p-ERK1/2 (phosphorylated-extracellular signal-regulated kinase 1/2). In summary, these AS-related signatures were closely related to cancer metastasis and survival and could be potential prognostic and therapeutic targets in CRC.

We obtained the immune landscape for the infiltration of six immune cell types in patients with CRC, classified samples according to TII risk score, and evaluated the infiltration level of different inflammatory cells. B cells, CD4T cells, dendritic cells, macrophages, and neutrophils were closely related to CRC prognosis. However, there was no significant difference in CD8T cell composition between the two study groups. Tumor-infiltrating immune cells are associated with tumor progression and invasion (53). Immunotherapy is revolutionizing the treatment of advanced cancer (54). By comprehensive research of immune-related DEGs and immune cell profiles in CRC carcinogenesis, we identified new therapeutic targets to improve responses to CRC immunotherapy.

Our results showed that AS likely altered the TII level. Deeper research on both risk models demonstrated that in all seven AS types, compared with the low-AS-risk and low-TII-risk groups, the high-AS-risk and high-TII-risk groups had poor prognoses in CRC. However, different AS types were associated with different TII risk characteristics. To identify the correlation between AS events and TII cell distribution, we used the TIMER database to determine the immune landscape of the infiltration of 6 immune cell types in CRC. Our results showed that AA and AP events directly affected the concentration of CD4T cells, and the level of CD8T cells was closely correlated with AT and ES events. However, the other AS events showed relatively limited effects. CD4T and CD8T cells in a CRC immune microenvironment were not dominated by AS, and the other four immune cell types, including B, dendritic, macrophage, and neutrophil cells strongly correlated with AS events.

We performed a Cox multivariate analysis to develop the AS and TII risk score model to identify and validate AS-related DEGs according to the AS risk scoring system and proved the connection between AS risk and prognosis in patients with CRC. We conducted a two-factor survival analysis according to patient survival outcomes and evaluated the correlation between TII and different AS events. The selected genes could provide novel signatures for targeting AS signal pathways and immune regulation for CRC immunotherapy. Unlike traditional studies on CRC bio-markers, we analyzed a large number of clinical samples and elucidated the relationship between the regulation of AS-related genes and the incidence and prognosis of CRC, while also focusing on the effect of different AS events on CRC-TII characteristics. With the use of queues in the TCGA database, we identified and validated AS-related signatures and further assessed their clinical risks. This study also used the TIMER database to construct an assessment model of AS-TII cell, which improved the efficiency of research. However, because this study relies on fitting, its clinical accuracy needs to be further assessed in cohort studies. The selected signatures require further clinical study to confirm their function in the CRC process.

## Conclusion

In this study, we used data from public databases to establish and assess AS and TII risk models, and elucidate the association between specific AS and immune signatures and patient outcome in CRC. Furthermore, we performed in-depth mechanism analyses by examining the TII cell distribution in different AS event types, thereby confirming biological relationships among the AS and TII landscapes. The signatures that we identified are potential prognostic and therapeutic targets. Thus, our results could form a basis for future experimental and clinical studies to develop novel prognostics and therapeutic strategies for CRC. Furthermore, we established a robust method for the in-depth study of CRC etiology and progression, which could also be used to study other cancers.

## Data Availability Statement

The datasets presented in this study can be found in online repositories. The names of the repository/repositories and accession number(s) can be found in the article/**Supplementary Material**.

## Author Contributions

J-yS conceived and designed the study. B-fY provided administrative support. Q-fY undertook the provision of study materials or patients. Y-yB collected and assembled data. DT and D-nW undertook data analysis and interpretation. All authors contributed to the article and approved the submitted version. All authors are accountable for the content of the work.

## Funding

This study received support from the Natural Science Foundation of Liaoning Science and Technology Department (0180550645) and Scientific research funding project of Liaoning Provincial Department of education (ky2019-08).

## Conflict of Interest

The authors declare that the research was conducted in the absence of any commercial or financial relationships that could be construed as a potential conflict of interest.

## Supplementary Material

Supplementary Figure 1 The differentially expressed genes (DEGs) of survival-associated AS events between the CRC and normal groups. **(A)** Volcano Plot was drawn to show the DEAS identified in CRC. The red points in the plot represent DEGs related to AS with statistical significance (*P*-value < 0.05, |log FC|) ≥ 2). The 20 most significant signatures in seven AS types were **(B)** AA **(C)** AD **(D)** AP **(E)** AT **(F)** ES **(G)** ME **(H)** RI.

Supplementary Figure 2 Functional enrichment analysis results of DEGs related to AS in CRC. Significantly enriched Kyoto Encyclopedia of Genes and Genomes pathways of differentially splicing genes in CRC are shown as **(A)** bar plot, **(B)** bubble plot, and **(C, D)** Circle plot. Significantly enriched Gene Ontology of differentially splicing genes in CRC illustrated in **(A)** bar plot, **(B)** bubble plot, and **(C, D)** Circle plot.

Supplementary Figure 3 Identification of hub AS signature in CRC. Cox regression and LASSO regression were conducted for the evaluation of the hub AS associated signature in different AS events. A-H represents the AS events AA, AD, AP, AT, ES, ME, RI, and total events, respectively.

Supplementary Figure 4 Identification of hub immune signature in CRC. **(A)** Volcano plot showing the DEGs in CRC tissue versus normal samples. **(B)** The differential expression of identified hub immune signature in tumor and normal groups are shown in a heatmap plot. **(C, D)** LASSO regression was conducted to search the hub tumor-associated immune signature. **(E)** Signatures selected for the establishment of the risk scoring mode base on the Multivariate Cox regression analysis results.
