# Supplementary figures and images for: Alternative Splicing Events in Tumor Immune Infiltration in Colorectal Cancer
Source: Front Oncol. 2021 Apr 29;11:583547. doi: 10.3389/fonc.2021.583547 (PMC8117221; doi:10.3389/fonc.2021.583547)

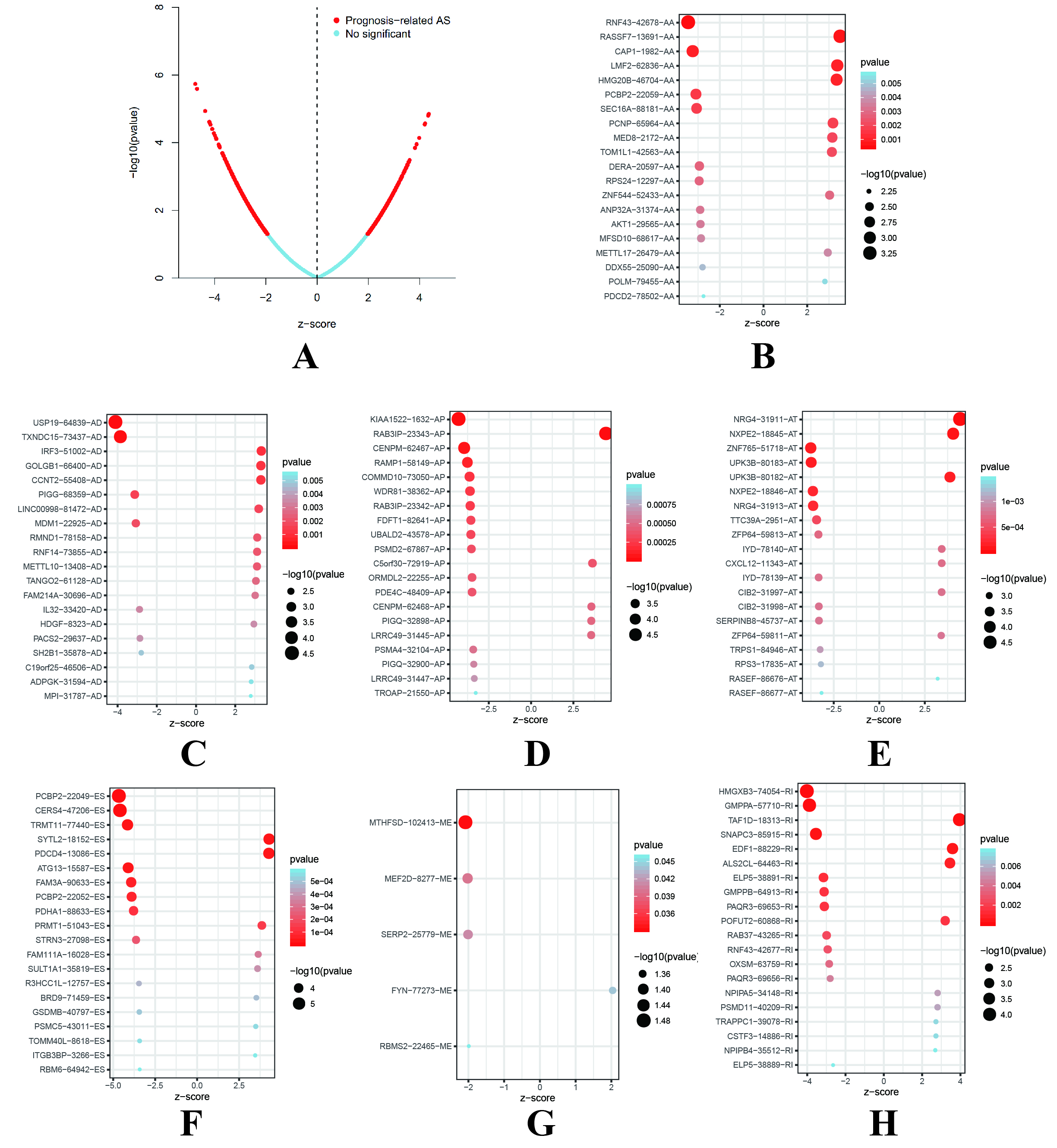

Supplement: Supplementary Figure 1 — The differentially expressed genes (DEGs) of survival-associated AS events between the CRC and normal groups. (A) Volcano Plot was drawn to show the DEAS identified in CRC. The red points in the plot represent DEGs related to AS with statistical significance (P-value < 0.05, |log FC|) ≥ 2). The 20 most significant signatures in seven AS types were (B) AA (C) AD (D) AP (E) AT (F) ES (G) ME (H) RI. [file Image_1.jpeg]

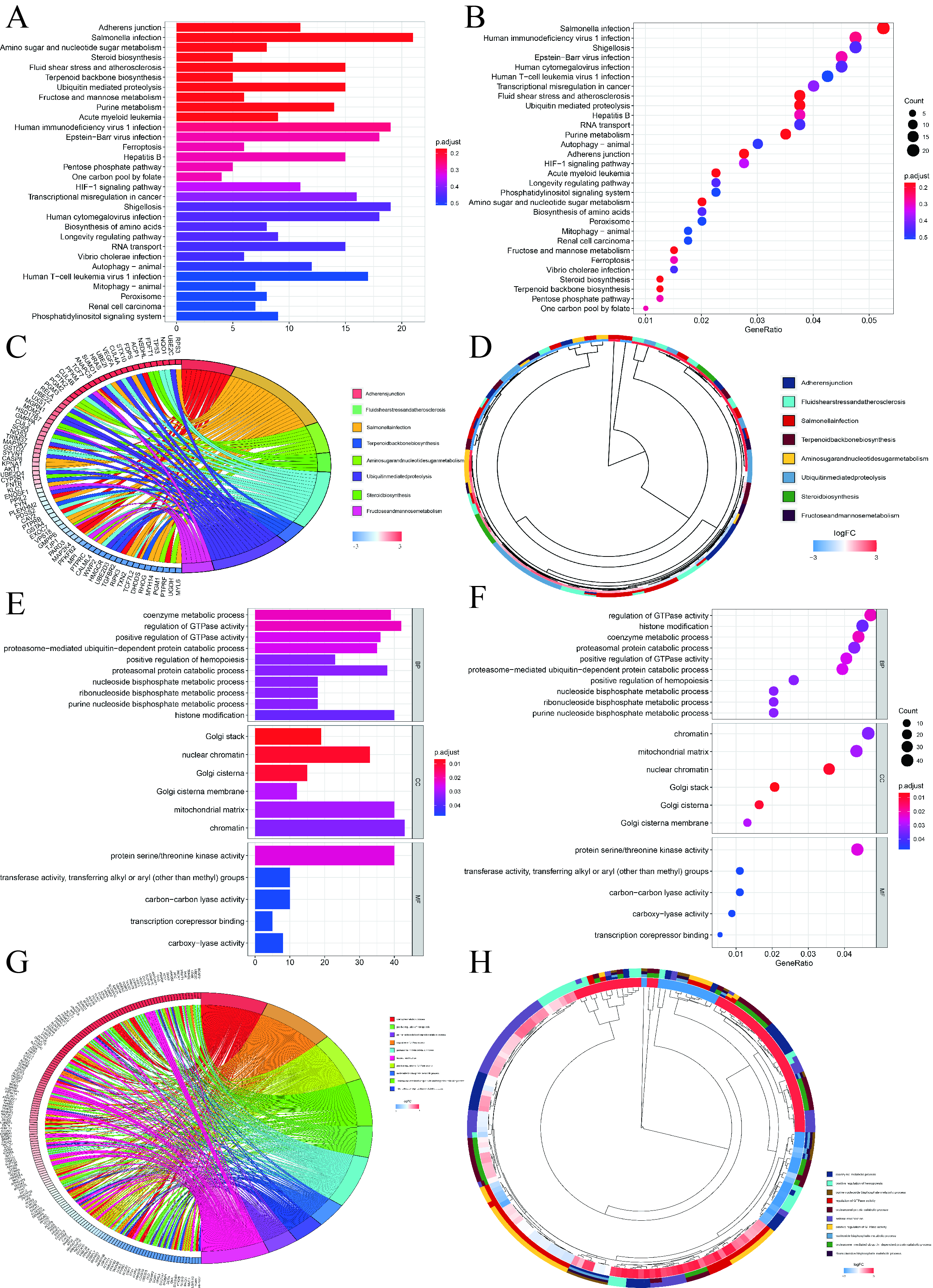

Supplement: Supplementary Figure 2 — Functional enrichment analysis results of DEGs related to AS in CRC. Significantly enriched Kyoto Encyclopedia of Genes and Genomes pathways of differentially splicing genes in CRC are shown as (A) bar plot, (B) bubble plot, and (C, D) Circle plot. Significantly enriched Gene Ontology of differentially splicing genes in CRC illustrated in (A) bar plot, (B) bubble plot, and (C, D) Circle plot. [file Image_2.jpeg]

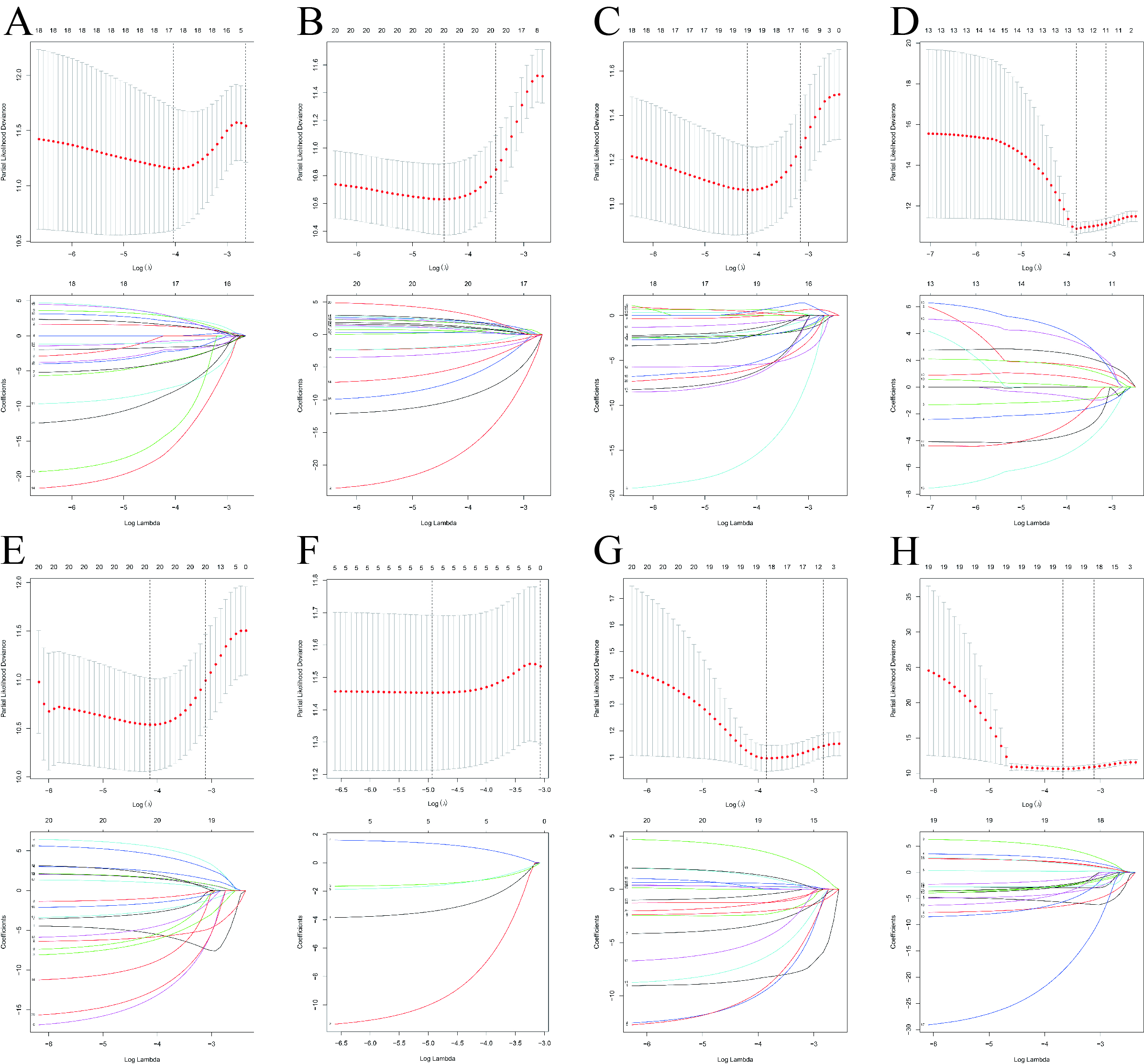

Supplement: Supplementary Figure 3 — Identification of hub AS signature in CRC. Cox regression and LASSO regression were conducted for the evaluation of the hub AS associated signature in different AS events. A-H represents the AS events AA, AD, AP, AT, ES, ME, RI, and total events, respectively. [file Image_3.jpeg]

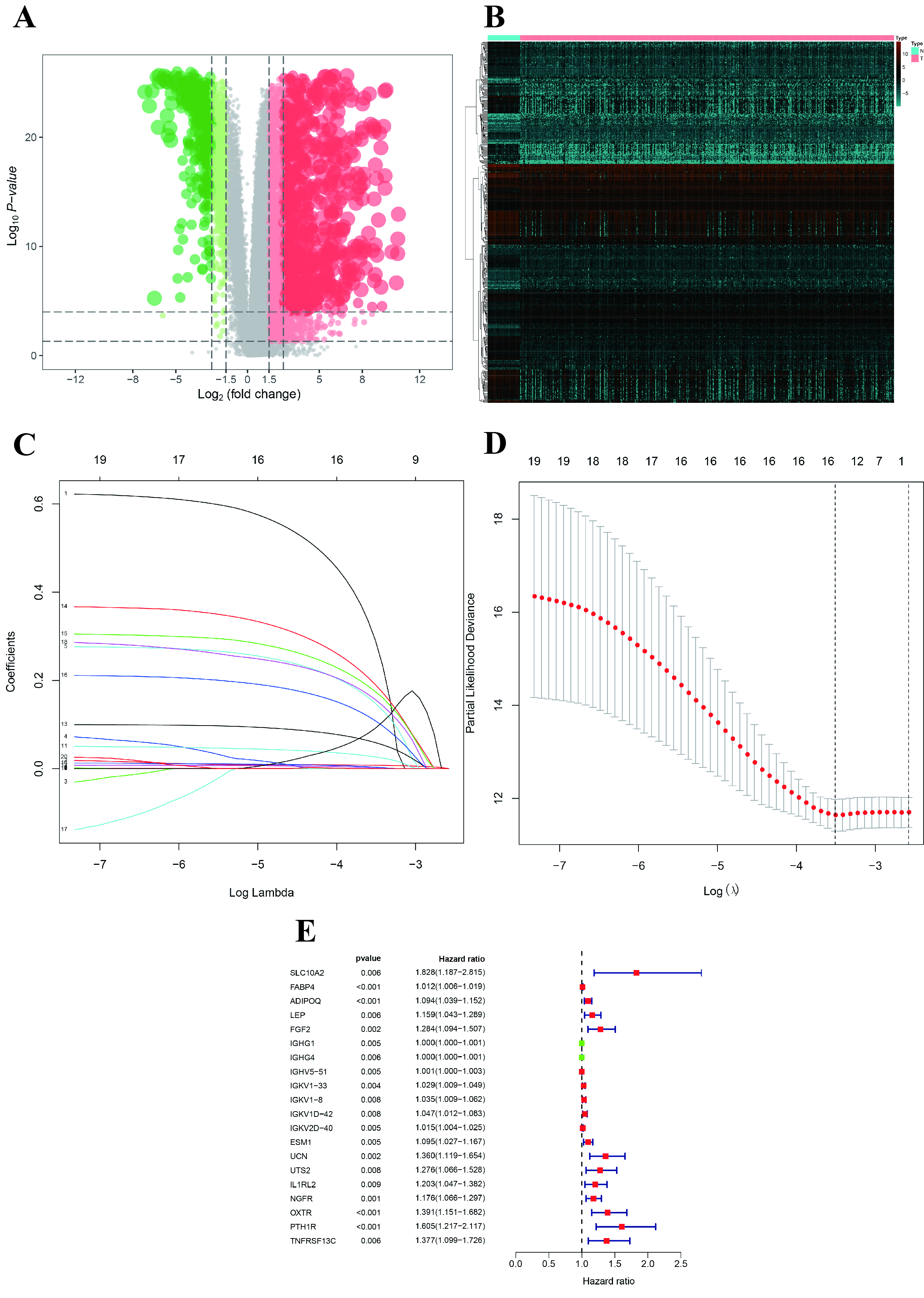

Supplement: Supplementary Figure 4 — Identification of hub immune signature in CRC. (A) Volcano plot showing the DEGs in CRC tissue versus normal samples. (B) The differential expression of identified hub immune signature in tumor and normal groups are shown in a heatmap plot. (C, D) LASSO regression was conducted to search the hub tumor-associated immune signature. (E) Signatures selected for the establishment of the risk scoring mode base on the Multivariate Cox regression analysis results. [file Image_4.jpeg]
