# Supplementary material for: Alternative Splicing Events in Tumor Immune Infiltration in Colorectal Cancer
Source: Front Oncol. 2021 Apr 29;11:583547. doi: 10.3389/fonc.2021.583547 (PMC8117221; doi:10.3389/fonc.2021.583547)
Supplement: Supplementary file 5 [file Table_1.docx]

Table.S1 The biological roles of AS related signatures in CRC

|  | ID | Description | pvalue | geneID |
| --- | --- | --- | --- | --- |
| KEGG | hsa04520 | Adherens junction | 0.000724 | ACP1/FYN/4LMO7/MAP3K7/PARD3/PTPRB/PTPRF/TCF7/TCF7L2/TGFBR2/TJP1 |
|  | hsa05132 | Salmonella infection | 0.002317 | AKT1/CASP8/EXOC7/HRAS/KLC1/KPNA1/MAP2K4/MAP3K7/MYH14/MYL6/PLEKHM2/PTPRC/RELA/RHOG/RIPK3/RPS3/STX10/TCF7/TCF7L2/TXN2/VPS18 |
|  | hsa00520 | Amino sugar and nucleotide sugar metabolism | 0.002363 | GMPPA/GMPPB/MPI/PGM1/PGM2/PGM3/UGDH/UXS1 |
|  | hsa00100 | Steroid biosynthesis | 0.002533 | CYP2R1/FDFT1/HSD17B7/NSDHL/SC5D |
|  | hsa05418 | Fluid shear stress and atherosclerosis | 0.003908 | AKT1/CALML4/CAV2/GSTA4/GSTO2/MAP2K4/MAP3K7/NOS3/NQO1/PTK2/RELA/SUMO1/TP53/TXN2/VEGFA |
|  | hsa00900 | Terpenoid backbone biosynthesis | 0.003961 | DHDDS/FDPS/FNTB/HMGCR/PDSS2 |
|  | hsa04120 | Ubiquitin mediated proteolysis | 0.004183 | ANAPC5/CUL2/CUL4A/CUL4B/MDM2/MGRN1/PPIL2/SYVN1/TRIM37/UBE2C/UBE2D3/UBE2D4/UBE2I/UBE2Z/WWP2 |
|  | hsa00051 | Fructose and mannose metabolism | 0.005281 | ENOSF1/GMPPA/GMPPB/MPI/PFKFB2/PFKM |
|  | hsa00230 | Purine metabolism | 0.005321 | ADA/AMPD3/ATIC/CANT1/ENTPD4/FHIT/GMPR2/IMPDH1/ITPA/NUDT5/PAPSS2/PDE4C/PGM1/PGM2 |
|  | hsa05221 | Acute myeloid leukemia | 0.005812 | AKT1/HRAS/PER2/RELA/RPS6KB1/RUNX1/STAT3/TCF7/TCF7L2 |
|  | hsa05170 | Human immunodeficiency virus 1 infection | 0.009646 | AKT1/AP1B1/AP1G2/APOBEC3D/CALML4/CASP8/CUL4A/CUL4B/HRAS/IRF3/LIMK2/MAP3K7/NFATC1/PAK4/PRKCA/PTK2/PTK2B/RELA/RPS6KB1 |
|  | hsa05169 | Epstein-Barr virus infection | 0.011676 | AKT1/BLNK/CASP8/CCND3/CD44/IRF3/MAP2K4/MAP3K7/MDM2/NCOR2/NFKB2/PSMC5/PSMD11/PSMD2/PSMD8/RELA/STAT3/TP53 |
|  | hsa04216 | Ferroptosis | 0.015332 | ACSL1/ATG5/GSS/LPCAT3/PCBP2/TP53 |
|  | hsa05161 | Hepatitis B | 0.015401 | AKT1/CASP10/CASP8/HRAS/IRF3/MAP2K4/MAP3K7/NFATC1/PRKCA/PTK2B/RELA/STAT3/TGFBR2/TP53/YWHAZ |
|  | hsa00030 | Pentosephosphate pathway | 0.015447 | DERA/PFKM/PGM1/PGM2/RPE |
|  | hsa00670 | One carbon pool by folate | 0.015772 | AMT/ATIC/MTHFD2L/SHMT1 |
|  | hsa04066 | HIF-1 signaling pathway | 0.019999 | AKT1/CUL2/ENO2/MKNK1/NOS3/PFKM/PRKCA/RELA/RPS6KB1/STAT3/VEGFA |
|  | hsa05202 | Transcriptional misregulation in cancer | 0.023711 | CCNT2/ETV7/GZMB/ITGB7/MAX/MDM2/NCOR1/NFKBIZ/PER2/PTK2/RELA/RUNX1/SUPT3H/TCF3/TGFBR2/TP53 |
|  | hsa05131 | Shigellosis | 0.030302 | AKT1/AKT1S1/ATG5/CBX3/CD44/GABARAPL1/ILK/IRF3/MALT1/MAP3K7/MDM2/PTK2/RBCK1/RELA/RPS6KB1/TP53/U2AF1L4/UBE2D3/UBE2D4 |
|  | hsa05163 | Human cytomegalovirus infection | 0.032603 | AKT1/CALML4/CASP8/CXCL12/GNA12/GNAS/HRAS/IRF3/MDM2/NFATC1/PRKCA/PTK2/PTK2B/RELA/RPS6KB1/STAT3/TP53/VEGFA |
|  | hsa01230 | Biosynthesis of amino acids | 0.032952 | ACO1/ASL/ENO2/GPT/MAT2A/PFKM/RPE/SHMT1 |
|  | hsa04211 | Longevity regulating pathway | 0.033193 | AKT1/AKT1S1/ATG13/ATG5/HRAS/RELA/RPS6KB1/TP53/ULK1 |
|  | hsa03013 | RNA transport | 0.035701 | ACIN1/DDX20/EIF2B1/EIF2B2/EIF4G3/FMR1/GEMIN8/MAGOHB/NUP85/POM121/PRMT5/RAN/SUMO1/THOC6/UBE2I |
|  | hsa05110 | Vibrio cholerae infection | 0.0373 | ATP6V0A2/ATP6V0B/GNAS/KDELR1/PRKCA/TJP1 |
|  | hsa04140 | Autophagy-animal | 0.041154 | AKT1/AKT1S1/ATG10/ATG13/ATG5/GABARAPL1/HRAS/MAP3K7/RPS6KB1/TANK/ULK1/WDR41 |
|  | hsa05166 | Human T-cell leukemia virus 1 infection | 0.047189 | AKT1/ANAPC5/CCND3/ETS1/FDPS/HRAS/IL15RA/MAP2K4/NFATC1/NFKB2/POLB/RAN/RANBP3/RELA/TCF3/TGFBR2/TP53 |
| BP | GO:0006732 | coenzyme metabolic process | 7.65E-06 | ACLY/ACOT11/ACOT7/ACOT8/ACSL1/ADPGK/ATIC/DERA/DGAT1/DLD/ELOVL5/ENO2/FLAD1/GSTO2/HMGCR/MAT2A/MTHFD2L/NCOR1/NMNAT1/NMNAT3/NUP85/OXSM/PDK3/PDPR/PDSS2/PDXK/PFKFB2/PFKM/PGM1/PGM2/POM121/PPCDC/RPE/SHMT1/SHPK/SLC25A32/STAT3/THEM4/TP53 |
|  | GO:0043087 | regulation of GTPase activity | 9.21E-06 | AGAP1/ALS2CL/ARAP1/ARFGAP1/ARHGAP10/ARHGAP29/ARHGAP8/ARHGEF10L/ARHGEF7/CAV2/CHN2/DENND1A/DEPDC5/DOCK10/DOCK7/ELMOD2/ELMOD3/EZH2/FAM13A/HRAS/ITGB1BP1/LIMS1/LLGL2/MAP4K4/MAPRE2/NGEF/PIP5K1A/PTK2/PTK2B/RABEP1/RABEP2/RANBP3/RGS10/RGS3/RHOG/RIC8A/SH3BP1/SPRY1/TBC1D2/TBC1D7/TMED2/TNK2 |
|  | GO:0043547 | positive regulation of GTPase activity | 1.83E-05 | AGAP1/ALS2CL/ARAP1/ARFGAP1/ARHGAP10/ARHGAP29/ARHGAP8/ARHGEF10L/ARHGEF7/CAV2/CHN2/DENND1A/DEPDC5/DOCK10/DOCK7/ELMOD2/ELMOD3/EZH2/FAM13A/HRAS/LIMS1/LLGL2/MAP4K4/MAPRE2/PIP5K1A/PTK2B/RABEP1/RABEP2/RANBP3/RGS10/RGS3/RHOG/RIC8A/SH3BP1/TBC1D2/TBC1D7 |
|  | GO:0043161 | proteasome-mediated ubiquitin-dependent protein catabolic process | 2.15E-05 | AKT1/ANAPC5/ATXN3/BCAP31/BFAR/CSNK1E/CUL4A/DERL3/DNAJC10/EDEM2/FHIT/GBA/GNA12/HFE/MDM2/PCBP2/PCNP/PSMA4/PSMC5/PSMD11/PSMD2/PSMD8/RBCK1/RNF4/SUMO1/SYVN1/TMUB1/TMUB2/TRIB2/TRIM13/UBE2C/UBE2D3/UBXN4/USP19/WWP2 |
|  | GO:1903708 | positive regulation of hemopoiesis | 4.20E-05 | ACIN1/ACVR1B/ADA/ADAM8/ANKRD54/CASP8/CD46/ETS1/GNAS/HMGB2/LGALS9/MALT1/MDK/MYB/NFKBIZ/PDCD2/PRKCA/PRMT1/PTPRC/RUNX1/STAT3/TGFBR2/ZBTB46 |
|  | GO:0010498 | proteasomal protein catabolic process | 4.63E-05 | AKT1/ANAPC5/ATXN3/BCAP31/BFAR/CSNK1E/CUL4A/CUL4B/DERL3/DNAJC10/EDEM2/FHIT/FMR1/GBA/GNA12/HFE/MDM2/OSBPL7/PCBP2/PCNP/PSMA4/PSMC5/PSMD11/PSMD2/PSMD8/RBCK1/RNF4/SUMO1/SYVN1/TMUB1/TMUB2/TRIB2/TRIM13/UBE2C/UBE2D3/UBXN4/USP19/WWP2 |
|  | GO:0033865 | nucleoside bisphosphate metabolic process | 6.30E-05 | ABHD14B/ACLY/ACOT11/ACOT7/ACOT8/ACSL1/DGAT1/DLD/ELOVL5/HMGCR/OXSM/PAPSS2/PDK3/PDPR/PPCDC/SLC35B3/SULT1A1/THEM4 |
|  | GO:0033875 | ribonucleoside bisphosphate metabolic process | 6.30E-05 | ABHD14B/ACLY/ACOT11/ACOT7/ACOT8/ACSL1/DGAT1/DLD/ELOVL5/HMGCR/OXSM/PAPSS2/PDK3/PDPR/PPCDC/SLC35B3/SULT1A1/THEM4 |
|  | GO:0034032 | purine nucleoside bisphosphate metabolic process | 6.30E-05 | ABHD14B/ACLY/ACOT11/ACOT7/ACOT8/ACSL1/DGAT1/DLD/ELOVL5/HMGCR/OXSM/PAPSS2/PDK3/PDPR/PPCDC/SLC35B3/SULT1A1/THEM4 |
|  | GO:0016570 | histone modification | 7.77E-05 | ARID4B/ATG5/ATXN3/AUTS2/BRD8/BRPF1/COPRS/CUL4B/EED/EPC2/EZH2/FMR1/HAT1/HCFC1/HDAC6/KANSL2/KDM5A/MAP3K7/MUC1/MYB/PAXBP1/PCGF3/PER2/PIH1D1/PRDM4/PRKCA/PRMT1/PRMT5/SETD6/SETMAR/SIRT3/SUPT3H/SUV39H2/TADA2A/TADA2B/TET2/TP53/TRIM37/VEGFA/WDR5 |
| CC | GO:0005795 | Golgi stack | 1.24E-05 | ARAP1/ARSE/BCAP31/CANT1/COG2/FUT3/GOLGA3/GOLPH3L/GPR89A/HID1/RAB34/SAR1B/SORT1/ST3GAL4/SULF2/TMED2/TMEM59/TOM1L1/UXS1 |
|  | GO:0000790 | nuclear chromatin | 3.38E-05 | APTX/BRD8/BRD9/CBX3/CPSF6/DDX11/EED/ESCO2/EZH2/GATAD2A/HAT1/HMGB2/INO80/MAX/MUC1/MXD1/NASP/NCAPD3/NCOR1/NCOR2/NFATC1/PHF12/POLR3GL/RELA/STAT3/TCF12/TCF3/TCF7/TCF7L2/TNKS1BP1/TP53/TRPS1/ZNF385A |
|  | GO:0031985 | Golgi cisterna | 5.44E-05 | ARAP1/BCAP31/CANT1/FUT3/GOLGA3/GOLPH3L/GPR89A/HID1/RAB34/SAR1B/SORT1/ST3GAL4/TMED2/TMEM59/UXS1 |
|  | GO:0032580 | Golgi cisterna membrane | 0.000216 | ARAP1/BCAP31/CANT1/FUT3/GOLGA3/GOLPH3L/GPR89A/SAR1B/SORT1/ST3GAL4/TMED2/UXS1 |
|  | GO:0005759 | mitochondrial matrix | 0.000284 | ALAS1/AMT/ATXN3/AUH/CLPX/DARS2/DECR1/DLD/ETFDH/FASTK/FDXR/FLAD1/GUF1/HARS2/ISCA1/KARS/MRPL20/MRPL27/MRPL33/MRPL52/MRPS22/MRPS7/MTHFD2L/NDUFA7/NDUFS7/NMNAT3/PDK3/PDPR/PDSS2/RPS3/RPUSD3/SIRT3/STYXL1/TBRG4/TEFM/THEM4/TOP1MT/TP53/TRIT1/TXN2 |
|  | GO:0000785 | chromatin | 0.000382 | APTX/BRD8/BRD9/CBX3/CPSF6/DDX11/EED/ESCO2/EXOSC3/EZH2/FAM111A/GATAD2A/HAT1/HMGB2/HMGN1/HMGN3/INO80/IST1/MAX/MUC1/MXD1/NASP/NCAPD3/NCOR1/NCOR2/NFATC1/PHF12/POLR3GL/RAD21/RAN/RELA/RRP8/STAG1/STAT3/SUV39H2/TCF12/TCF3/TCF7/TCF7L2/TNKS1BP1/TP53/TRPS1/ZNF385A |
| MF | GO:0004674 | protein serine/threonine kinase activity | 3.27E-05 | ACVR1B/ADCK5/AKT1/CCND3/CDK10/CDK11A/CDK13/CDK8/CLK1/CSNK1E/FASTK/GAK/HIPK1/ILK/LIMK2/LRRK1/MAP3K11/MAP3K7/MAP4K3/MAP4K4/MAP4K5/MARK2/MINK1/MKNK1/MOK/NEK3/NEK4/NEK6/OBSCN/PAK4/PASK/PDK3/PKMYT1/PRKCA/PTK2B/RIPK3/STK3/TGFBR2/TNK2/ULK1 |
|  | GO:0016765 | transferase activity, transferring alkyl or aryl (other than methyl) groups | 0.000267 | DHDDS/DPH2/FNTB/GSTA4/GSTO2/HMBS/MAT2A/PDSS2/RABGGTA/TRIT1 |
|  | GO:0016830 | carbon-carbon lyase activity | 0.000267 | CSAD/DDC/DDT/DERA/ECHDC1/PDXDC1/PPCDC/SHMT1/UMPS/UXS1 |
|  | GO:0001222 | transcription corepressor binding | 0.000278 | FAM89B/HDGF/NEK6/PHF12/SUMO1 |
|  | GO:0016831 | carboxy-lyase activity | 0.000317 | CSAD/DDC/DDT/ECHDC1/PDXDC1/PPCDC/UMPS/UXS1 |
