# Supplementary material for: Alternative Splicing Events in Tumor Immune Infiltration in Colorectal Cancer
Source: Front Oncol. 2021 Apr 29;11:583547. doi: 10.3389/fonc.2021.583547 (PMC8117221; doi:10.3389/fonc.2021.583547)
Supplement: Supplementary file 6 [file Table_2.docx]

TABLE. S2 The detailed information of seven alternative splicing events singatures related to overall survival in CRC patients (n=433).

| Events | id | coef | HR | HR.95L | pvalue |
| --- | --- | --- | --- | --- | --- |
| AA | RNF43\|42678\|AA | -2.14074 | 0.117568 | 0.012789 | 0.058579 |
|  | RASSF7\|13691\|AA | 1.740185 | 5.698398 | 1.26542 | 0.023416 |
|  | CAP1\|1982\|AA | -5.4945 | 0.004109 | 5.78E-05 | 0.011564 |
|  | HMG20B\|46704\|AA | 4.305759 | 74.12546 | 1.161293 | 0.042308 |
|  | SEC16A\|88181\|AA | -6.35297 | 0.001742 | 3.77E-05 | 0.001163 |
|  | MED8\|2172\|AA | 4.382541 | 80.04116 | 0.434249 | 0.099647 |
|  | DERA\|20597\|AA | -11.0411 | 1.60E-05 | 4.47E-11 | 0.090641 |
|  | RPS24\|12297\|AA | -1.04235 | 0.352624 | 0.089384 | 0.136605 |
|  | ZNF544\|52433\|AA | 2.303565 | 10.0098 | 0.972803 | 0.052772 |
|  | ANP32A\|31374\|AA | -26.1928 | 4.21E-12 | 2.56E-21 | 0.015554 |
|  | AKT1\|29565\|AA | -20 | 2.06E-09 | 1.16E-14 | 0.001181 |
|  | POLM\|79455\|AA | 4.973801 | 144.5754 | 1.090184 | 0.046088 |
|  | PDCD2\|78502\|AA | -13.6544 | 1.17E-06 | 1.07E-10 | 0.004004 |
| AD | USP19\|64839\|AD | -11.8636 | 7.04E-06 | 4.65E-08 | 3.64E-06 |
|  | TXNDC15\|73437\|AD | -18.7816 | 6.97E-09 | 5.78E-15 | 0.008566 |
|  | GOLGB1\|66400\|AD | 2.924184 | 18.61904 | 0.739677 | 0.07561 |
|  | CCNT2\|55408\|AD | 1.732781 | 5.656362 | 1.200889 | 0.028417 |
|  | PIGG\|68359\|AD | -4.47956 | 0.011338 | 0.000437 | 0.007024 |
|  | LINC00998\|81472\|AD | 3.562016 | 35.23416 | 1.355462 | 0.032118 |
|  | MDM1\|22925\|AD | -2.60342 | 0.07402 | 0.021645 | 3.33E-05 |
|  | RMND1\|78158\|AD | 2.954709 | 19.19614 | 0.493874 | 0.113605 |
|  | METTL10\|13408\|AD | 3.291806 | 26.89138 | 3.098016 | 0.002831 |
|  | TANGO2\|61128\|AD | 2.582373 | 13.22849 | 3.068267 | 0.000533 |
|  | IL32\|33420\|AD | -7.9965 | 0.000337 | 1.59E-06 | 0.003426 |
|  | PACS2\|29637\|AD | -11.5122 | 1.00E-05 | 4.74E-08 | 2.49E-05 |
|  | ADPGK\|31594\|AD | 2.030375 | 7.616942 | 1.466717 | 0.015706 |
|  | MPI\|31787\|AD | 6.20064 | 493.0644 | 3.849597 | 0.012266 |
| AP | KIAA1522\|1632\|AP | -2.46802 | 0.084752 | 0.009938 | 0.024021 |
|  | CENPM\|62467\|AP | -3.33959 | 0.035451 | 0.003117 | 0.007101 |
|  | RAMP1\|58149\|AP | -3.06669 | 0.046575 | 0.0044 | 0.010851 |
|  | COMMD10\|73050\|AP | -19.3195 | 4.07E-09 | 8.24E-14 | 0.000459 |
|  | WDR81\|38362\|AP | -8.58743 | 0.000186 | 1.97E-07 | 0.014056 |
|  | RAB3IP\|23342\|AP | -2.77684 | 0.062235 | 0.003259 | 0.064997 |
|  | PDE4C\|48409\|AP | -8.70427 | 0.000166 | 2.87E-08 | 0.048853 |
|  | PSMA4\|32104\|AP | -2.78147 | 0.061947 | 0.00163 | 0.133965 |
|  | PIGQ\|32900\|AP | -2.31118 | 0.099144 | 0.010759 | 0.041381 |
|  | LRRC49\|31447\|AP | -3.63262 | 0.026447 | 0.002383 | 0.003092 |
|  | TROAP\|21550\|AP | -10.3822 | 3.10E-05 | 4.40E-09 | 0.02164 |
| AT | NRG4\|31911\|AT | 3.03857 | 20.87538 | 3.840449 | 0.000435 |
|  | NXPE2\|18845\|AT | 2.365123 | 10.64535 | 3.060537 | 0.0002 |
|  | UPK3B\|80183\|AT | -2.36809 | 0.09366 | 0.02251 | 0.001132 |
|  | CXCL12\|11343\|AT | 2.165477 | 8.718762 | 2.350231 | 0.001206 |
|  | CIB2\|31997\|AT | 6.07675 | 435.6111 | 7.32881 | 0.00355 |
|  | SERPINB8\|45737\|AT | -8.23456 | 0.000265 | 3.55E-07 | 0.014714 |
|  | ZFP64\|59811\|AT | 6.029024 | 415.3095 | 7.400689 | 0.003346 |
|  | TRPS1\|84946\|AT | -4.02314 | 0.017897 | 0.000747 | 0.013059 |
|  | RPS3\|17835\|AT | -5.21836 | 0.005416 | 4.05E-05 | 0.03669 |
| ES | CERS4\|47206\|ES | -6.54587 | 0.001436 | 4.33E-05 | 0.000248 |
|  | TRMT11\|77440\|ES | -9.04387 | 0.000118 | 4.48E-07 | 0.001476 |
|  | SYTL2\|18152\|ES | 3.091064 | 22.00047 | 2.3429 | 0.00683 |
|  | PDCD4\|13086\|ES | 6.633173 | 759.8891 | 6.112482 | 0.007025 |
|  | ATG13\|15587\|ES | -18.2153 | 1.23E-08 | 5.84E-11 | 2.46E-11 |
|  | FAM3A\|90633\|ES | -4.22516 | 0.014623 | 0.001695 | 0.000122 |
|  | PCBP2\|22052\|ES | -2.12749 | 0.119136 | 0.016919 | 0.032653 |
|  | PDHA1\|88633\|ES | -8.61837 | 0.000181 | 5.28E-06 | 1.74E-06 |
|  | PRMT1\|51043\|ES | 6.148783 | 468.1472 | 4.512921 | 0.009425 |
|  | STRN3\|27098\|ES | -3.66228 | 0.025674 | 0.004148 | 8.22E-05 |
|  | FAM111A\|16028\|ES | 2.138853 | 8.489693 | 2.307655 | 0.00129 |
|  | SULT1A1\|35819\|ES | 2.098168 | 8.151224 | 0.996496 | 0.050384 |
|  | R3HCC1L\|12757\|ES | -10.8545 | 1.93E-05 | 5.04E-09 | 0.00992 |
|  | BRD9\|71459\|ES | 2.260224 | 9.585238 | 2.798751 | 0.00032 |
|  | GSDMB\|40797\|ES | -2.55002 | 0.07808 | 0.008553 | 0.023821 |
|  | TOMM40L\|8618\|ES | -6.29714 | 0.001842 | 2.60E-05 | 0.003754 |
|  | ITGB3BP\|3266\|ES | 3.346287 | 28.39711 | 4.018553 | 0.000796 |
|  | RBM6\|64942\|ES | -16.0511 | 1.07E-07 | 1.23E-10 | 3.35E-06 |
| ME | MTHFSD\|102413\|ME | -3.95579 | 0.019144 | 0.000644 | 0.022266 |
|  | MEF2D\|8277\|ME | -11.7125 | 8.19E-06 | 4.99E-12 | 0.108713 |
|  | SERP2\|25779\|ME | -1.67561 | 0.187194 | 0.023457 | 0.113833 |
|  | FYN\|77273\|ME | 1.645048 | 5.181257 | 1.173359 | 0.029935 |
|  | RBMS2\|22465\|ME | -1.92925 | 0.145258 | 0.01492 | 0.096612 |
| RI | HMGXB3\|74054\|RI | -14.3156 | 6.06E-07 | 1.07E-10 | 0.001163 |
|  | GMPPA\|57710\|RI | -2.53055 | 0.079615 | 0.007479 | 0.03599 |
|  | TAF1D\|18313\|RI | 5.76143 | 317.8026 | 10.62808 | 0.00089 |
|  | SNAPC3\|85915\|RI | -14.6507 | 4.34E-07 | 7.36E-10 | 6.76E-06 |
|  | ELP5\|38891\|RI | -5.33758 | 0.004807 | 0.00022 | 0.000696 |
|  | GMPPB\|64913\|RI | -12.9902 | 2.28E-06 | 4.74E-10 | 0.002676 |
|  | PAQR3\|69653\|RI | -4.86008 | 0.00775 | 9.48E-05 | 0.03055 |
|  | RAB37\|43265\|RI | -9.49853 | 7.50E-05 | 1.10E-06 | 1.04E-05 |
|  | RNF43\|42677\|RI | -8.53279 | 0.000197 | 2.36E-07 | 0.012921 |
|  | NPIPB4\|35512\|RI | 2.326404 | 10.24105 | 1.533598 | 0.016334 |
|  | ELP5\|38889\|RI | -2.15296 | 0.11614 | 0.018531 | 0.021496 |
| Total | CERS4\|47206\|ES | -8.08285 | 0.000309 | 9.16E-06 | 6.68E-06 |
|  | KIAA1522\|1632\|AP | -3.92992 | 0.019645 | 0.002482 | 0.000197 |
|  | NRG4\|31911\|AT | 3.575889 | 35.72635 | 6.778206 | 2.48E-05 |
|  | TRMT11\|77440\|ES | -4.91561 | 0.007331 | 1.90E-05 | 0.105679 |
|  | SYTL2\|18152\|ES | 2.470688 | 11.83058 | 1.525671 | 0.01807 |
|  | PDCD4\|13086\|ES | 7.147434 | 1270.841 | 9.111728 | 0.004554 |
|  | ATG13\|15587\|ES | -12.2627 | 4.72E-06 | 1.89E-08 | 1.35E-05 |
|  | CENPM\|62467\|AP | -2.49954 | 0.082123 | 0.007683 | 0.03866 |
|  | FAM3A\|90633\|ES | -5.09419 | 0.006132 | 0.000724 | 2.97E-06 |
|  | NXPE2\|18845\|AT | 2.754013 | 15.70552 | 4.726751 | 6.95E-06 |
|  | GMPPA\|57710\|RI | -4.69256 | 0.009163 | 0.001125 | 1.16E-05 |
|  | TXNDC15\|73437\|AD | -30.6333 | 4.97E-14 | 5.11E-20 | 1.33E-05 |
|  | TAF1D\|18313\|RI | 3.509817 | 33.44215 | 1.485397 | 0.027175 |
|  | PDHA1\|88633\|ES | -7.69077 | 0.000457 | 1.50E-05 | 1.01E-05 |
|  | RAMP1\|58149\|AP | -4.11878 | 0.016264 | 0.002098 | 8.08E-05 |
