# Supplementary material for: Alternative Splicing Events in Tumor Immune Infiltration in Colorectal Cancer
Source: Front Oncol. 2021 Apr 29;11:583547. doi: 10.3389/fonc.2021.583547 (PMC8117221; doi:10.3389/fonc.2021.583547)
Supplement: Supplementary file 7 [file Table_3.docx]

TABLE. S3 The detailed information of TII singatures related to overall survival in CRC patients (n=391).

| ID | coef | HR | HR.95L | pvalue |
| --- | --- | --- | --- | --- |
| SLC10A2 | 0.641722 | 1.89975 | 1.164246 | 0.010209 |
| FABP4 | 0.010718 | 1.010775 | 1.003911 | 0.002051 |
| FGF2 | 0.289903 | 1.336298 | 1.109899 | 0.002207 |
| IGHG4 | 0.000399 | 1.000399 | 1.000078 | 0.014713 |
| IGKV1D-42 | 0.0513 | 1.052638 | 1.014563 | 0.00635 |
| IGKV2D-40 | 0.01091 | 1.010969 | 0.999797 | 0.054337 |
| ESM1 | 0.103754 | 1.109327 | 1.042198 | 0.001123 |
| UCN | 0.361421 | 1.435367 | 1.161902 | 0.000804 |
| UTS2 | 0.294475 | 1.342422 | 1.107449 | 0.002704 |
| IL1RL2 | 0.204652 | 1.227098 | 1.063359 | 0.0051 |
| OXTR | 0.262402 | 1.30005 | 1.027432 | 0.028864 |
